# Dietary aromatic amino acids, their food sources, and incident hypertension in Chinese adults: a prospective cohort study

**DOI:** 10.3389/fnut.2026.1956118

**Published:** 2026-10-22

**Authors:** Ming Gong, Haiying Gao, Huiying Yu, Wenlong Wu, Xue Gao, Yingzhen Bu, Xiangpeng Kong, Xin Zhao

**Affiliations:** 1 Department of Cardiology, The Second Hospital of Dalian Medical University, Dalian, China; 2 Department of General Medicine, The Second Hospital of Dalian Medical University, Dalian, China; 3 Laboratory of Basic Medicine, General Hospital of Northern Theater Command, Shenyang, China; 4 The First Clinical College, Liaoning University of Traditional Chinese Medicine, Shenyang, China

**Keywords:** aromatic amino acids, China Health and Nutrition Survey, food sources, hypertension, phenylalanine, prospective cohort, tryptophan, tyrosine

## Abstract

**Background:**

Prospective evidence linking dietary aromatic amino acids (AAAs), including phenylalanine (Phe), tyrosine (Tyr), and tryptophan (Trp), to incident hypertension currently remains limited, particularly regarding their individual components and food sources. Herein, we examined the association between dietary AAAs, their food sources, and incident hypertension among Chinese adults.

**Methods:**

Using the China Health and Nutrition Survey database, this prospective cohort study collected data from 10,087 adults without hypertension at baseline. Baseline dietary intake was assessed using three consecutive 24-h dietary recalls, combined with household food weighing; the energy-adjusted AAA intake was expressed in g/1,000 kcal. Incident hypertension was defined as measured blood pressure, physician diagnosis, or use of antihypertensive medication. Mixed-effects Cox proportional hazards models with household identifiers as random intercepts were used to estimate hazard ratios (HRs) and 95% confidence intervals (CIs). Dose–response, food-source-specific, subgroup, and sensitivity analyses were performed.

**Results:**

During the 95,153 person-years of follow-up, 3,531 participants developed hypertension. In fully adjusted models, the highest quartile of total AAA intake was associated with a modestly higher risk of incident hypertension compared with the lowest quartile (HR, 1.16; 95% CI, 1.03–1.31; P for trend = 0.006). Positive associations were more consistent for Phe (HR for Q4 vs. Q1, 1.26; 95% CI, 1.11–1.44) and Trp (HR, 1.29; 95% CI, 1.14–1.46) individually, while Tyr intake showed no significant association with hypertension. Dose–response analyses indicated approximately linear positive associations for total AAA, Phe, and Trp. The association for total AAA was attenuated following additional adjustment for baseline province (HR for Q4 vs. Q1, 1.10; 95% CI, 0.97–1.24), while the positive associations for Phe and Trp persisted. Cereals were the predominant contributors to AAA intake, and plant-derived Phe and Trp were positively associated with the risk of hypertension.

**Conclusion:**

Higher dietary intakes of Phe and Trp showed consistent positive associations with incident hypertension, while Tyr showed no clear association. The modest association for total AAA was attenuated after adjusting for baseline province and should therefore be interpreted cautiously in relation to potential regional confounding factors. These findings support the component-specific interpretation of dietary AAAs and consideration of their regional and food source contexts.

## 1 Introduction

Hypertension is a leading modifiable risk factor for cardiovascular and kidney diseases worldwide, and remains a major public health burden in China (1, 2). Dietary modification is central to hypertension prevention, with clinical trials establishing the blood pressure–lowering effects of dietary patterns emphasizing fruit, vegetables, low-fat dairy products, sodium reduction, and broader lifestyle modifications (3, 4). Recent metabolomic analyses of the DASH and DASH-Sodium feeding trials further identified several amino acid–related metabolites associated with blood pressure reductions during dietary interventions, including tryptophan betaine, N-methylglutamate, and proline derivatives (5). However, beyond established dietary factors, such as sodium, potassium, and overall diet quality, the role of protein-related components and amino acid profiles in the development of hypertension remains incompletely understood.

Aromatic amino acids (AAAs), including phenylalanine (Phe), tyrosine (Tyr), and tryptophan (Trp), are biologically relevant and exert distinct metabolic functions. Phe is an essential amino acid and precursor of Tyr, which is involved in catecholamine synthesis; conversely, Trp is used to synthesize serotonin, kynurenine pathway metabolites, and gut microbiota-derived indole compounds (6, 7). In addition, the microbial co-metabolism of AAAs, including the conversion of Phe to phenylacetylglutamine, has been linked to cardiovascular risk (8, 9). AAAs may be related to vascular tone, endothelial function, sympathetic activity, inflammation, oxidative stress, and insulin sensitivity through both host and microbial pathways, all of which are relevant to blood pressure regulation.

Prospective evidence regarding the association between dietary AAA intake and hypertension remains limited and heterogeneous. The Tehran Lipid and Glucose Study reported that a higher dietary AAA intake was associated with an increased risk of incident hypertension, with this association being most evident for Phe (10). A recent systematic review and meta-analysis further suggested an unfavorable association between higher dietary amino acid intake, including AAAs, and hypertension risk, while emphasizing the differences in population characteristics, dietary assessment, exposure definitions, and covariate adjustment across studies (11). In contrast, studies investigating circulating amino acids have yielded mixed findings, partly because circulating concentrations reflect not only dietary intake, but also endogenous metabolism, renal handling, insulin resistance, and gut microbial activity (12). Moreover, previous studies have rarely examined individual AAA components, dose–response patterns, repeated dietary exposure, or food source-specific associations within the same prospective framework.

The food source context may be particularly important, as amino acids are consumed within foods and dietary patterns, rather than as isolated nutrients. Notably, the same amino acids may be embedded in food matrices with distinct quantities of carbohydrates, fat, fiber, sodium, potassium, and other bioactive compounds (13). In Chinese adults, plant-derived amino acids may largely reflect cereal-dominant dietary patterns, rather than plant-rich dietary patterns dominated by vegetables, fruits, legumes, nuts, and whole grains. Conversely, animal-derived amino acids may originate from heterogeneous food sources, including red and processed meat, poultry, fish, eggs, dairy products, and offal. Although emerging evidence suggests that amino acid–health associations may differ by dietary source, source-specific evidence for AAAs remains scarce (14).

To address these gaps, we used data from the China Health and Nutrition Survey, a long-term prospective cohort with repeated dietary assessments, to examine the association between energy-adjusted total AAA intake, its individual components, and incident hypertension among Chinese adults. We further evaluated the dose–response relationships, food source-specific associations, potential population heterogeneity, and robustness across multiple sensitivity analyses. We hypothesized that higher dietary AAA intake would be associated with incident hypertension, and that these associations may differ across individual AAA components and dietary sources.

## 2 Methods

### 2.1 Study population

This prospective cohort study used data from the China Health and Nutrition Survey (CHNS), an ongoing household-based open cohort study initiated in 1989, to examine the population-level changes in nutrition and health during China’s social and economic transformation. The CHNS uses multistage random cluster sampling to collect participants in urban and rural communities. The survey waves used for dietary exposure assessment covered nine provinces; Beijing, Shanghai, and Chongqing were added to three municipalities in 2011 (15, 16). In the present study, dietary exposure was assessed using the 1997, 2000, 2004, 2006, 2009, and 2011 survey waves, with incident hypertension was monitored through to 2015. Dietary data from the 2015 survey were not used as the raw dietary records were not fully available.

Participants who entered the analytic cohort during their first eligible survey wave between 1997 and 2011 were considered eligible. The inclusion criteria were: age ≥18 years, complete 3-day dietary data, plausible total energy intake, available baseline hypertension information, no hypertension or pregnancy at baseline, and at least one subsequent assessment of hypertension status. Of the 28,978 participants with at least one dietary assessment, we sequentially excluded 3,705 with incomplete 3-day dietary data, 6,062 aged <18 years, 947 with implausible total energy intake (defined as <800 or >4,200 kcal/day for men and <600 or >3,500 kcal/day for women), and 292 who were pregnant. Among the remaining 17,972 adults, 28 with missing baseline hypertension information, 4,746 with prevalent hypertension, and 3,111 with missing follow-up data after the baseline were additionally excluded. The final analytic cohort comprised 10,087 adults. A flowchart of participant selection is shown in Figure 1.

**Figure 1:**
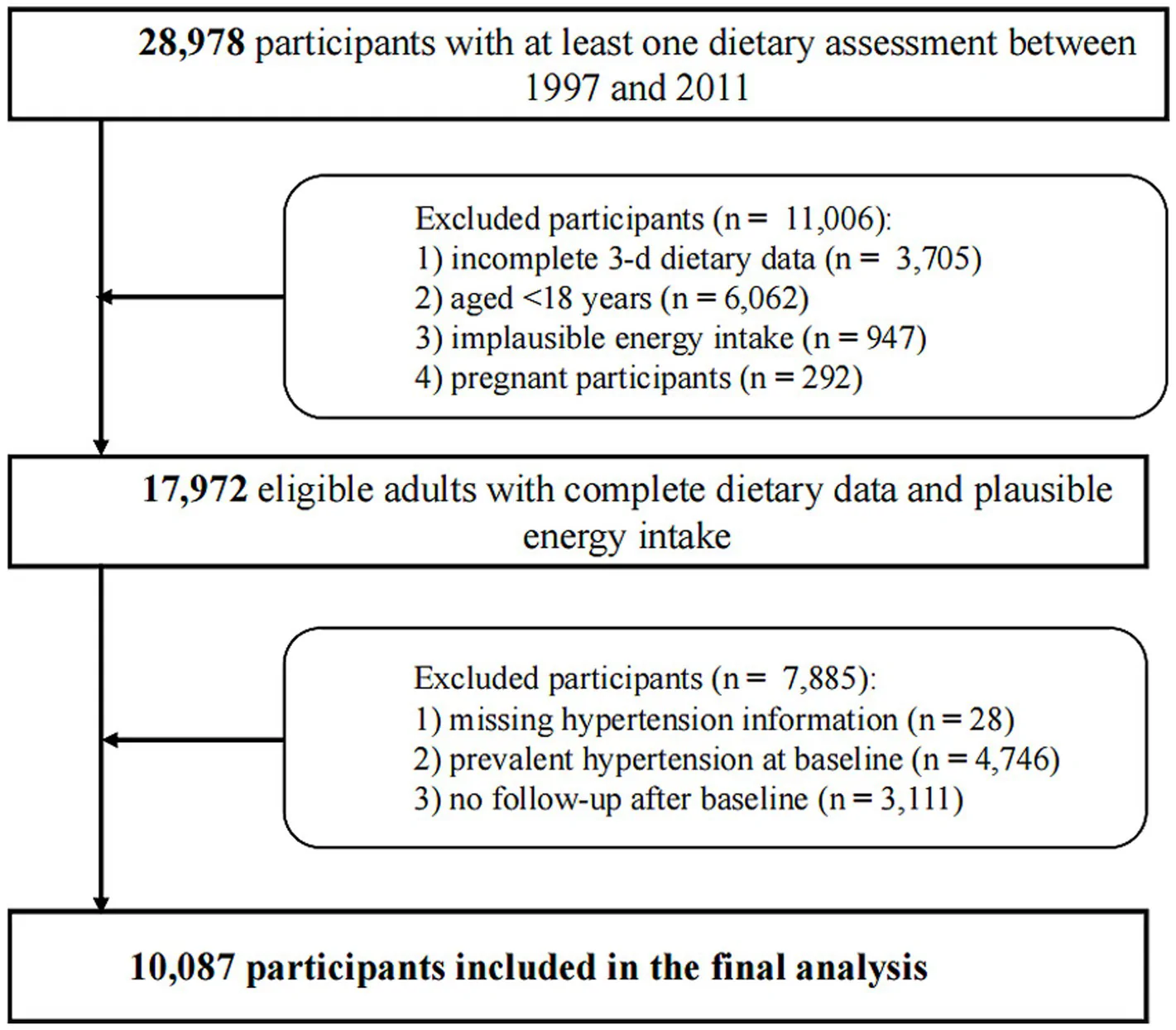
Flow diagram of participant selection.

The CHNS was approved by the institutional review boards of the University of North Carolina at Chapel Hill and the National Institute for Nutrition and Health, Chinese Center for Disease Control and Prevention. Written informed consent to participate was obtained from all participants.

### 2.2 Dietary assessment and aromatic amino acid intake

Dietary intake was assessed using 3 consecutive 24-h dietary recalls, combined with a household food inventory during the same 3 day period. Trained interviewers collected detailed information on all foods and beverages consumed at and away from home. Household food consumption, including the consumption of edible oils and condiments, was assessed using a weighing and inventory-based method.

Daily nutrient intake was calculated using Chinese Food Composition Tables corresponding to each survey wave. Phe, Tyr, and Trp intake was estimated for each participant, with AAA intake defined as the sum of the Phe, Tyr, and Trp intakes. To account for differences in total energy intake, AAA and its components were energy-adjusted using the nutrient density method, with values expressed as g/1000 kcal. Baseline dietary intake, defined as the first eligible dietary assessment, was used as the primary exposure in the main analyses. In the sensitivity analyses, the cumulative average intake was updated using all available dietary assessments obtained prior to the beginning of each risk interval; when no new dietary assessment was available, the most recently-calculated cumulative average was retained. The primary exposures were total AAA, Phe, Tyr, and Trp consumptions, analyzed as quartiles and continuous variables per 1-standard-deviation (SD) increment.

When Phe, Tyr, or Trp composition values were unavailable, missing values were estimated at the food composition level from the arithmetic mean of non-missing values among foods within the same food code category and harmonized major food groups in the corresponding Chinese Food Composition Table. Food-code harmonization and food group classification were manually reviewed and replacement values were programmatically calculated. Rather than being assigned values according to a presumed dominant ingredient, mixed/other foods were processed using the same category- and food-group-based rules. The participant-level amino acid intake was not statistically significant. Further details are provided in the Supplementary methods.

For food source analyses, foods were classified into plant-, animal-, or mixed/other-derived sources, based on the harmonized food groups. Plant-derived sources included cereals and grains, soybeans and legumes, nuts and seeds, vegetables, fruits, tubers, and starches, while animal-derived sources included red and processed meat, poultry, fish and seafood, animal offal, eggs, milk, and dairy products. Mixed dishes and foods that could not be assigned exclusively to either plant- or animal-derived sources were classified as mixed/other-derived and were not split proportionally. Source-specific amino acid intake was calculated by summing the amino acid contributions of all foods within each source category.

### 2.3 Ascertainment of incident hypertension

Trained healthcare workers measured blood pressure using a standardized protocol for each survey wave. Systolic blood pressure (SBP) and diastolic blood pressure (DBP) were measured three times on the right arm using a mercury sphygmomanometer after the participants had rested in a seated position for at least 10 min; the average of the three measurements was used for analysis. Incident hypertension was defined as the first occurrence of any of the following during follow-up: systolic blood pressure ≥140 mmHg, diastolic blood pressure ≥90 mmHg, self-reported physician diagnosis of hypertension, or current use of antihypertensive medication (17). Participants with hypertension at baseline were excluded from the analysis. Person-years of follow-up were calculated as the time from baseline to the first occurrence of hypertension, the last survey with available hypertension information, or the end of follow-up in 2015, whichever occurred first. Deaths between survey waves were not separately identified in this analysis; in the absence of any observed hypertension event, the follow-up ended at the last available survey.

### 2.4 Covariate assessment

Information on demographic characteristics, socioeconomic status, lifestyle factors, medical history, and anthropometric measurements was collected using standardized questionnaires and physical examinations. Covariates included age, sex, residence, education level, income level, smoking status, alcohol consumption, physical activity level, baseline systolic blood pressure, body mass index (BMI) category, abdominal obesity, diabetes, myocardial infarction, stroke, total energy intake, sodium intake, and potassium intake. Residence was categorized as either urban or rural. Education level was categorized as no formal education, ≤9 y, or >9 y, according to the years of schooling. Annual net individual income was converted to 2015 constant Chinese Yuan using the survey wave-specific inflation index provided by the CHNS. The following income tertiles were defined using common cutpoints derived from the pooled distribution of inflation-adjusted income at the participants’ first eligible observations, and were applied consistently across different survey waves: <3,663 yuan, 3,663 to <11,625.06 yuan, and ≥11,625.06 yuan for the low-, middle-, and high-income groups, respectively. Smoking status was classified as never or ever smoked, while alcohol drinking was classified as non-drinker or drinker according to self-reported alcohol consumption over the preceding year. Physical activity was assessed using the CHNS questionnaire on occupational, transportation, domestic, and leisure-time activities. For each activity, the reported duration was converted to hours per week and multiplied by its assigned metabolic equivalent of task (MET) value; this value was subsequently summed across the activities to obtain the total physical activity in MET hours per week. Participants were classified as having low, moderate, or high physical activity, according to the tertiles of total physical activity within their baseline survey wave (18). BMI was calculated as the weight in kilograms divided by the height in meters squared (kg/m^2^) and was categorized as underweight (<18.5), normal weight (18.5–23.9), overweight (24.0–27.9), or obesity (≥28.0), according to the standard Chinese adult criteria (19). Abdominal obesity was defined as waist circumference ≥90 cm for men and ≥85 cm for women (19). Baseline systolic blood pressure was calculated as the average of three measurements obtained after the participants rested for at least 10 min. Baseline diabetes was defined as a self-reported history of physician-diagnosed diabetes or current use of glucose-lowering medications, collected using the information available at each participant’s first eligible survey wave. For participants whose baseline survey was conducted in 2009, available fasting blood glucose ≥7.0 mmol/L or HbA1c ≥ 6.5% was additionally used to identify diabetes (20). Information from subsequent survey waves was not used to classify baseline diabetes and missing biomarker measurements were not imputed. History of myocardial infarction and stroke was based on self-reported physician diagnoses. Dietary covariates, including total energy, sodium, and potassium intake, were estimated from the dietary assessment described above. Before imputation, we calculate the number and proportion of missing observations for each baseline covariate. For covariates with missing values, we applied separate univariate logistic regression models to examine whether missingness was associated with total AAA, Phe, Tyr, or Trp intake (per 1-SD increment) or incident hypertension status. Missing baseline covariates were handled using multiple imputations by chained equations, while the extent of missingness and its association with dietary AAA intake and incident hypertension status before imputation are reported in Supplementary Table S10.

### 2.5 Statistical analysis

Continuous and categorical variables are presented as the means ± SDs and numbers and percentages, respectively. Baseline characteristics are summarized according to extreme quartiles of total aromatic amino acid intake and its components in the main text, with full quartile distributions presented in the Supplementary material. Person-years were calculated from baseline to incident hypertension, the last available follow-up, or 2015, whichever occurred first. Associations between AAA intake and incident hypertension were examined using mixed-effects Cox proportional hazards models with household identifiers included as random intercepts. Exposure-specific and global Schoenfeld residual tests showed no evidence of proportional hazards violations for total AAA, Phe, Tyr, or Trp (Supplementary Table S11). Hazard ratios (HRs) and 95% confidence intervals (CIs) were estimated across the quartiles of energy-adjusted intake, with the lowest quartile (Q1) set as the reference and per 1-SD increment. Linear trends across quartiles were tested by assigning participants the median intake value of their corresponding quartile and modeling this value as a continuous variable.

Three sequential models were used in this study. Model 1 was adjusted for age, sex, urban residence, income, and educational levels; Model 2 was further adjusted for diabetes, myocardial infarction, stroke, smoking status, alcohol consumption, baseline systolic blood pressure, BMI, abdominal obesity, and physical activity level; and Model 3 was further adjusted for total energy, sodium, and potassium intake and was prespecified as the primary model. Restricted cubic spline analyses based on Model 3 were conducted to evaluate dose–response associations and potential nonlinearity. Spline curves were plotted across exposure values between the 5th and 95th percentiles to reduce any instability in the distribution tails. Overall associations and nonlinearities were assessed using likelihood ratio tests.

For food source analyses, source-specific intake was examined as plant-, animal-, and mixed/other-derived. Single-source models were fitted separately for each source-specific exposure, while mutual models included plant- and animal-derived intakes simultaneously. Interactions between plant- and animal-derived intakes were assessed by adding a product term between the centered and standardized source-specific intakes to Model 3 for each amino acid, with the results pooled across 20 imputed datasets. Mixed/other-derived intakes were not included in the mutual models owing to their small contributions and heterogeneous compositions. Predefined subgroup analyses were conducted according to age, sex, residence, income level, education level, smoking status, alcohol consumption, overweight/obesity status, abdominal obesity, and physical activity level. Multiplicative interactions were assessed using cross-product terms between the exposure and subgroup variables included in Model 3. Robustness was evaluated using complete-case analysis; exclusion of early events within the first 2 years; exclusion of participants with baseline diabetes, myocardial infarction, or stroke; additional adjustment for total protein intake, cumulative average intakes, and alternative exposure categorizations; and time-stratified analyses to assess the consistency of associations across different follow-up periods. Further sensitivity analysis included the baseline province as a categorical covariate to Model 3, while retaining urban/rural residence.

Missing baseline covariates were handled using multiple imputations with chained equations under a missing-at-random assumption. Overall, 20 imputed datasets were generated and the estimates were pooled using Rubin’s rules. Analyses were conducted using R software, version 4.5.2 (R Foundation for Statistical Computing). Statistical significance was set at a 2-sided *p* value <0.05.

## 3 Results

### 3.1 Study population and dietary aromatic amino acid intake

The final analysis included 10,087 adults without hypertension at baseline. Among the 10,087 participants, 6,252 (62.0%) underwent at least two eligible dietary assessments, with a mean of 2.81 assessments per participant (SD = 1.83; range = 1–6). The mean (SD) age was 42.6 (14.1) years, and 54.3% of participants were female. At the end of individual follow-up, the estimated mean (SD) age was 52.0 (14.4) years (range, 20–100 years). During the 95,153 person-years of follow-up, 3,531 incident hypertension cases were documented, corresponding to a crude incidence rate of 37.1 per 1,000 person-years. Baseline characteristics according to the quartiles of total AAA, Phe, Tyr, and Trp intake are presented in Supplementary Table S1. Participants with a higher total AAA intake were more likely to be female, reside in urban areas, have higher income and education levels, have a higher BMI, and have a greater prevalence of abdominal obesity and diabetes. They were less likely to smoke or drink alcohol, whereas baseline blood pressure levels were generally comparable across the quartiles. Similar patterns were observed for Phe, Tyr and Trp.

### 3.2 Aromatic amino acid components and incident hypertension

In the fully adjusted model, higher total AAA intake was associated with a higher risk of incident hypertension (Figure 2; Supplementary Table S2). The HR comparing the highest and lowest quartiles was 1.16 (95% CI: 1.03, 1.31; P-trend = 0.006), while the HR per 1-SD increment was 1.05 (95% CI: 1.00, 1.09; *p* = 0.039). Among the individual components, positive associations were observed between Phe and Trp. The corresponding HRs comparing the highest and lowest quartiles were 1.26 (95% CI: 1.11, 1.44; P-trend < 0.001) for Phe and 1.29 (95% CI: 1.14, 1.46; P-trend < 0.001) for Trp. Similar positive associations were observed in continuous analyses, with HRs per 1-SD increment of 1.08 (95% CI: 1.04, 1.14; *p* < 0.001) and 1.08 (95% CI: 1.03, 1.13; *p* = 0.001), respectively. In contrast, Tyr intake showed no significant associations with incident hypertension after full adjustment, either comparing the highest with the lowest quartile (HR: 1.09; 95% CI: 0.98, 1.21; P-trend = 0.168) or the per 1-SD increment (HR: 1.00; 95% CI: 0.97, 1.04; *p* = 0.871).

**Figure 2:**
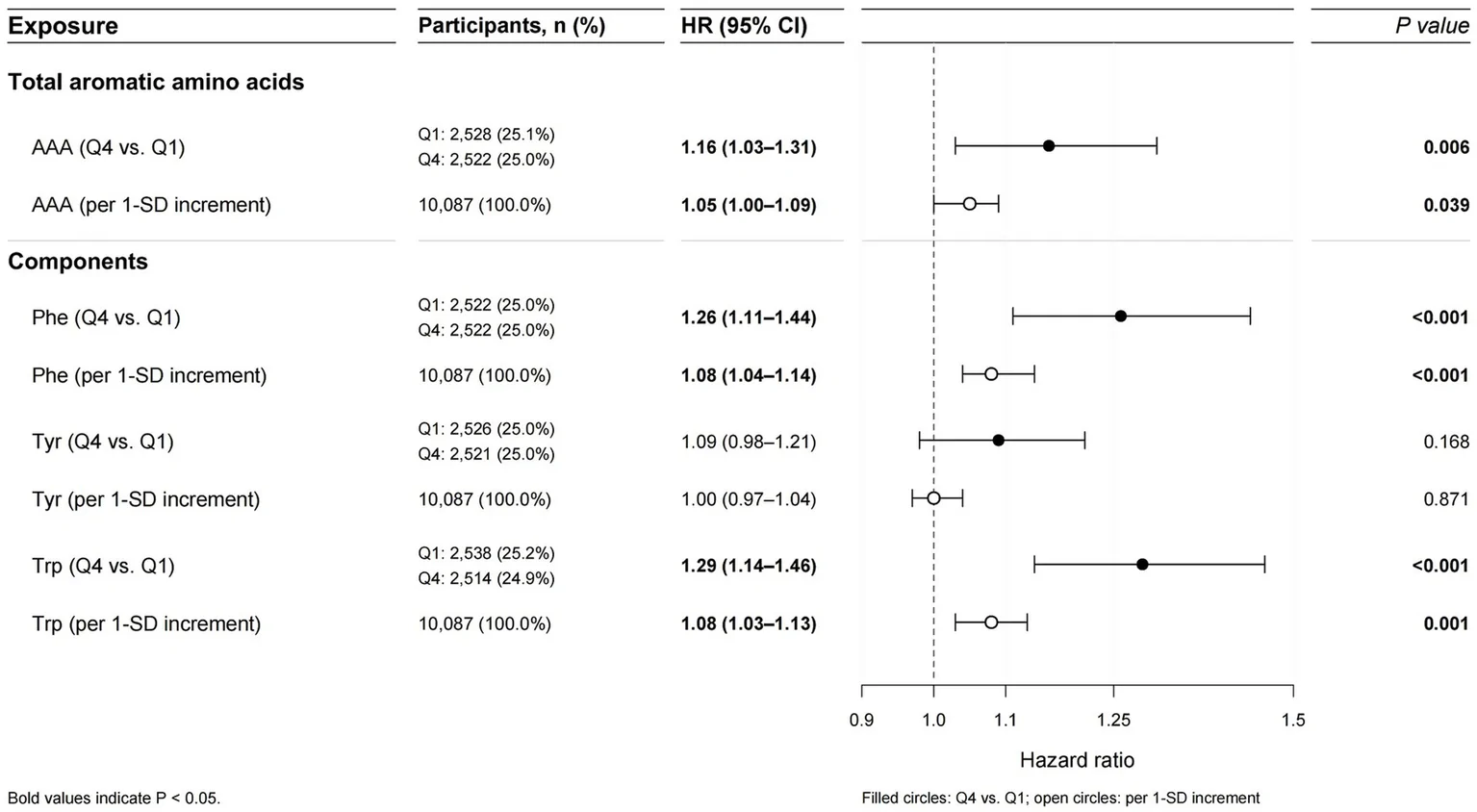
Associations between dietary aromatic amino acid intake and incident hypertension. Values shown are the adjusted hazard ratios (HRs) and 95% confidence intervals (CIs). For quartile comparisons, participant numbers are shown separately for Q1 and Q4; for per-1-SD analyses, the full analytical cohort was used. The percentages are based on data from 10,087 participants. The models included a household-level random intercept and were adjusted for age, sex, residence, income, education, diabetes, myocardial infarction, stroke, smoking, alcohol consumption, baseline systolic blood pressure, BMI, abdominal obesity, physical activity, total energy intake, sodium intake, and potassium intake. AAA, aromatic amino acid; BMI, body mass index; Phe, phenylalanine; SD, standard deviation; Trp, tryptophan; Tyr, tyrosine.

### 3.3 Dose–response patterns of dietary aromatic amino acids

Restricted cubic spline analyses suggested positive, approximately linear associations between incident hypertension and total AAA, Phe, and Trp intake (Figure 3). For total AAA intake, the overall association was statistically significant, with no evidence of nonlinearity (P-overall = 0.012; P-nonlinearity = 0.841). Similar patterns were observed for Phe (*p* < 0.001, *p* = 0.753) and Trp (*p* = 0.037, *p* = 0.363). In contrast, Tyr intake showed no significant dose–response association with incident hypertension (P-overall = 0.710; P-nonlinearity = 0.728).

**Figure 3:**
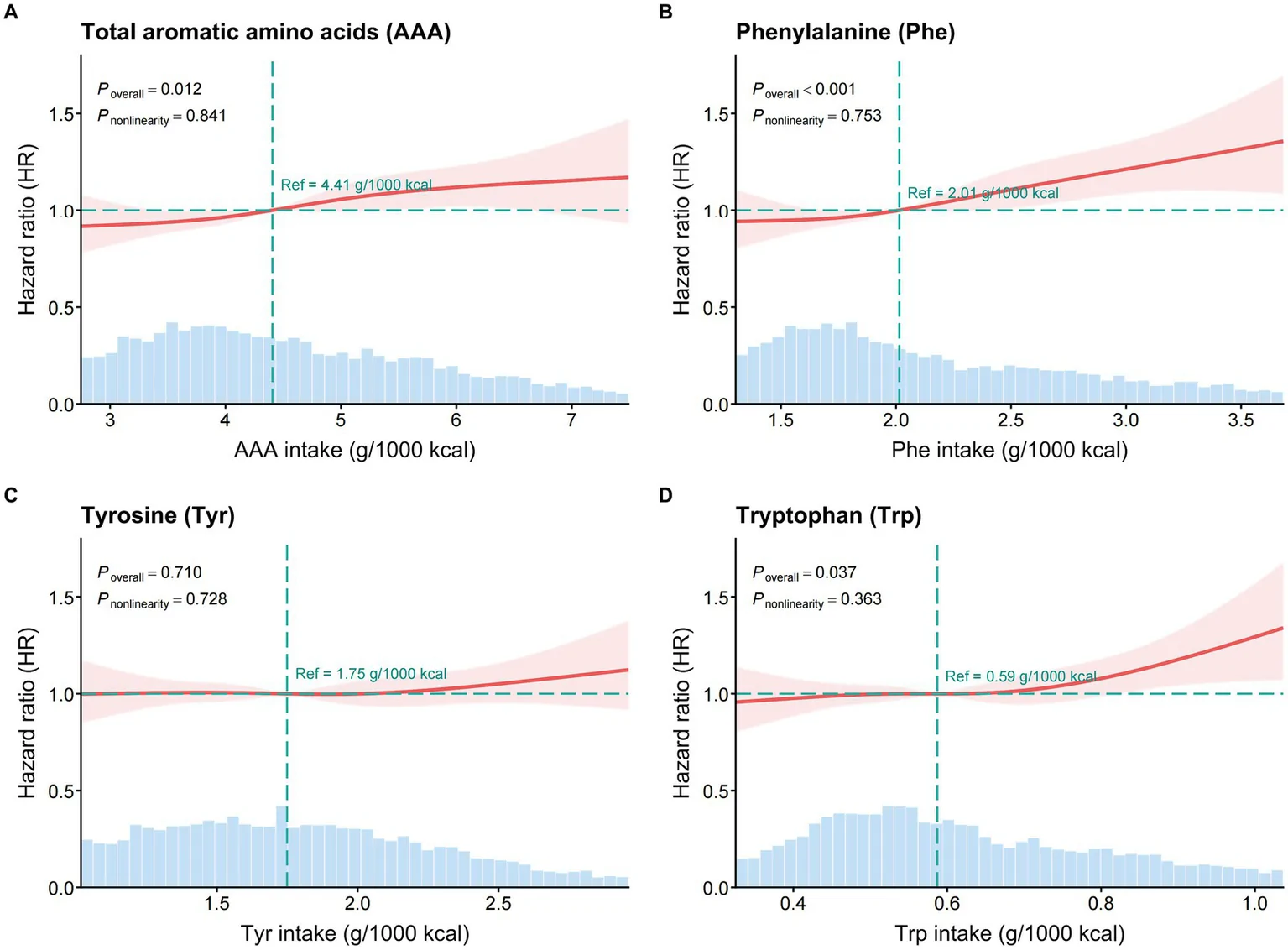
Dose–response associations between dietary aromatic amino acid intake and its components with incident hypertension. Restricted cubic spline curves are shown for **(A)** total AAAs, **(B)** Phe, **(C)** Tyr, and **(D)** Trp. Solid lines indicate adjusted hazard ratios (HRs), shaded regions indicate 95% confidence intervals (CIs), and histograms show intake distributions. The median intake was the reference (HR = 1); analyses were restricted to the 5th–95th percentiles. The models were adjusted for the covariates in Model 3 with a household-level random intercept. *p*-values for overall association and nonlinearity were obtained from likelihood ratio tests. AAA, aromatic amino acid; Phe, phenylalanine; Tyr, tyrosine; Trp, tryptophan.

### 3.4 Food sources of dietary aromatic amino acids and incident hypertension

Cereals were the predominant contributors to dietary AAA intake, accounting for 46.1% of the total AAA intake, followed by soybeans/legumes, red and processed meats, and tubers/starches (Figure 4). Similar source patterns were observed for Phe, Tyr, and Trp, although the relative contributions of specific food groups varied by component. When grouped by source type, plant-derived foods accounted for most of the total and component AAA intake, contributing to 73.5% of the total AAA, 73.9% of Phe, 74.3% of Tyr, and 70.0% of Trp intakes. The correlations between plant- and animal-derived AAA measurements were weak (Supplementary Figure S1).

**Figure 4:**
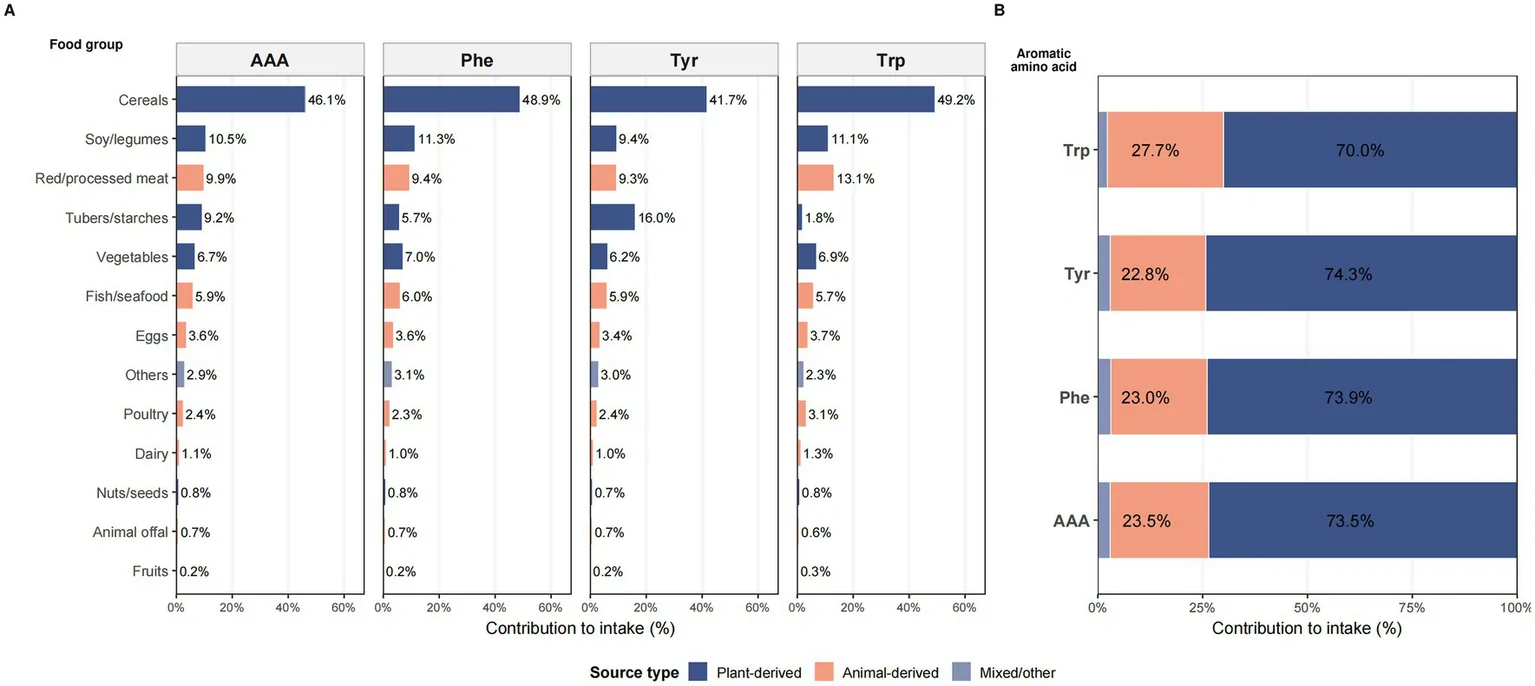
Major food sources of dietary aromatic amino acid intake. **(A)** Percentage contributions of major food groups to the total AAA, Phe, Tyr, and Trp intake. **(B)** Percentage contributions of plant-derived, animal-derived, and mixed/other-derived sources to each amino acid. Source categories are identified by the colors presented in the figure. AAA, aromatic amino acids; Phe, phenylalanine; Tyr, tyrosine; Trp, tryptophan.

In the mutually adjusted source-specific models, associations with incident hypertension differed according to the food source (Table 1). Plant-derived total AAA intake showed no significant association with hypertension risk, whereas plant-derived Phe and Trp intakes were positively associated with incident hypertension, with HRs per 1-SD increment of 1.06 (95% CI: 1.01, 1.10; *p* = 0.009) and 1.08 (95% CI: 1.03, 1.12; *p* = 0.001), respectively. Plant-derived tyrosine intake showed no significant association. In contrast, after mutual adjustment for plant-derived intake, animal-derived total AAA and their components were inversely associated with incident hypertension, with HRs per 1-SD increment ranging from 0.92 to 0.93. Single-source results were generally consistent for plant-derived Phe and Trp, and the crude and adjusted estimates for all source categories are presented in Supplementary Table S3. Fully adjusted animal-derived estimates were similar to those obtained from the mutually adjusted models. No clear evidence of any interaction between plant- and animal-derived intakes was observed for total AAA, Phe, Tyr, or Trp (P for interaction = 0.536, 0.771, 0.482, and 0.644, respectively; Supplementary Table S12).

**Table 1:** Source-specific aromatic amino acid intake and risk of incident hypertension in the mutually adjusted models.

Amino acid	Source	Participants with intake from this source	Contribution to total intake, %	HR (95% CI)	*p* value
AAA	Plant-derived	10,087 (100.0%)	73.5	1.03 (0.99–1.07)	0.195
AAA	Animal-derived	8,945 (88.7%)	23.5	0.92 (0.88–0.97)	**0.002**
Phe	Plant-derived	10,087 (100.0%)	73.9	1.06 (1.01–1.10)	**0.009**
Phe	Animal-derived	8,945 (88.7%)	23.0	0.93 (0.88–0.98)	**0.005**
Tyr	Plant-derived	10,087 (100.0%)	74.3	0.99 (0.95–1.02)	0.488
Tyr	Animal-derived	8,945 (88.7%)	22.8	0.92 (0.87–0.97)	**0.001**
Trp	Plant-derived	10,087 (100.0%)	70.0	1.08 (1.03–1.12)	**0.001**
Trp	Animal-derived	8,945 (88.7%)	27.7	0.93 (0.89–0.98)	**0.003**

Participants with intake from this source encompassed those with nonzero baseline intake; percentages were calculated for all 10,087 participants. The contribution to the total intake indicated the percentage of each amino acid supplied by the specified source. HRs (95% CIs) are presented per 1-SD increment of source-specific intake. Plant- and animal-derived intake of the same amino acids were included simultaneously in each model, with adjustments for the covariates specified in Model 3 and a household-level random intercept. Single-source results, including mixed/other-derived intakes, are presented in Supplementary Table S3. AAA, aromatic amino acid; CI, confidence interval; HR, hazard ratio; Phe, phenylalanine; SD, standard deviation; Trp, tryptophan; Tyr, tyrosine. Bold values indicate statistical significance (*p* < 0.05).

### 3.5 Robustness and population heterogeneity of the associations

In the prespecified subgroup analyses, the positive associations of total AAA, Phe, and Trp intake with incident hypertension were generally more pronounced among rural participants and those with lower educational attainment (Figure 5; Supplementary Table S4). Interaction tests additionally suggested heterogeneity in residence and education across several amino acid exposures. Additional evidence of effect modification was observed by sex for total AAA and Phe, and by age for Trp, while no consistent heterogeneity was observed according to income, smoking status, alcohol consumption, overweight/obesity, abdominal obesity, or physical activity. Tyr intake remained largely unassociated with the risk of hypertension in most subgroups.

**Figure 5:**
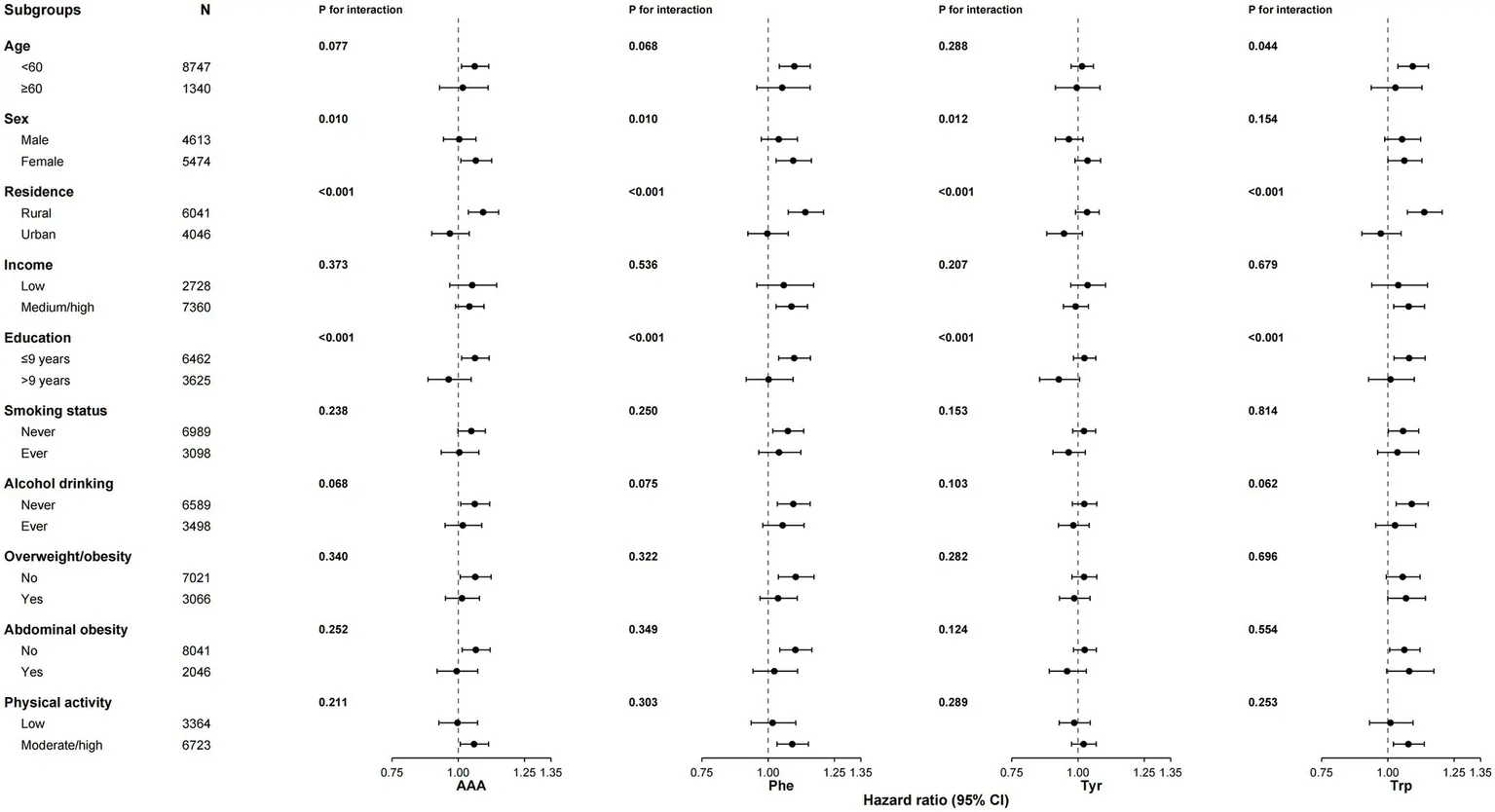
Subgroup analyses of dietary aromatic amino acid intake and incident hypertension. Points and horizontal lines indicate adjusted hazard ratios (HRs) and 95% confidence intervals (CIs) per 1-standard-deviation increment in total AAA, Phe, Tyr, and Trp intake. The models included a household-level random intercept and were adjusted for age, sex, residence, income, education, diabetes, myocardial infarction, stroke, smoking, alcohol consumption, baseline systolic blood pressure, BMI category, abdominal obesity, physical activity, total energy intake, sodium intake, and potassium intake, while excluding the corresponding subgroup variables. *p*-values for the interactions were obtained from exposure-by-subgroup terms. AAA, aromatic amino acid; BMI, body mass index; Phe, phenylalanine; Trp, tryptophan; Tyr, tyrosine.

The sensitivity analyses generally supported the component-specific pattern identified in the main analyses (Supplementary Tables S5–S9). When cumulative average intake was used, positive associations persisted for Phe and Trp, whereas the association for total AAA was attenuated, and Tyr showed no clear association. For total AAA, the HR per 1-SD increment was 1.04 (95% CI: 1.00, 1.08) with cumulative average intake, compared with 1.05 (95% CI: 1.00, 1.09) in the main analysis. The results were materially unchanged in complete-case analyses after excluding early hypertension cases or participants with baseline diabetes, myocardial infarction, or stroke, and with after further adjustment for total protein intake. Additional adjustment for baseline province attenuated the association for total AAA (HR for Q4 vs. Q1:1.10; 95% CI: 0.97, 1.24), while the positive associations for Phe and Trp persisted and Tyr showed no clear association (Supplementary Table S9). Analyses using tertiles and quintiles yielded patterns consistent with quartile analyses. Time-stratified analyses yielded directionally consistent, but less precise, estimates.

## 4 Discussion

In this prospective cohort study of Chinese adults initially free of hypertension, higher dietary Phe and Trp intakes showed the most consistent positive associations with incident hypertension, while Tyr intake showed no clear independent association. The association with total AAA was modest in the main analysis (HR for Q4 vs. Q1: 1.16; 95% CI, 1.03–1.31). Dose–response analyses supported approximately linear positive associations for total AAA, Phe, and Trp, while source-specific analyses showed that the associations were not uniform across different food sources. These findings highlight the importance of interpreting dietary AAAs in relation to their individual components and the food source context. The crude incidence rate of hypertension in our cohort was 37.1 per 1,000 person-years. This was higher than the 30.3 per 1,000 person-years calculated from the 4,329 cases and 142,868 person-years reported by Li and Shi (21), whose cohort had a mean baseline age of 40.2 years, but lower than the 45.0 per 1,000 person-years reported by Zhang et al. (22) in a CHNS cohort, with a mean baseline age of 41.2 years. Thus, our estimate falls between those of the two CHNS analyses of adults with broadly similar baseline ages, although differences in participant eligibility and survey waves used for dietary assessment should be considered when comparing these crude rates.

The total AAA association was attenuated after additional adjustment for baseline province (HR for Q4 vs. Q1, 1.10; 95% CI, 0.97–1.24), indicating sensitivity to regional adjustment. Differences in staple foods, dietary practices, socioeconomic circumstances, and other regional factors may have contributed to the main estimate, although province adjustment could not identify the responsible factors. The greater consistency in Phe and Trp findings across the analyses reinforces the importance of component-specific interpretations. Subgroup findings should be interpreted as exploratory. Differences by residence and education may further reflect variations in staple foods, overall dietary patterns, health literacy, or healthcare access; however, these explanations were not directly assessed. The modest attenuation of the total AAA estimate when the cumulative average intake was used may reflect dietary changes during follow-up or differences between baseline and updated exposure measures; however, these analyses cannot establish the reason for this attenuation.

### 4.1 Previous evidence and component-specific interpretation

Overall, our findings are broadly consistent with prior evidence suggesting that a higher dietary AAA intake may be associated with a greater hypertension risk, while extending this literature by highlighting the component- and source-specific heterogeneities. In the Tehran Lipid and Glucose Study, higher dietary AAA intake was prospectively associated with an increased risk of incident hypertension, with Phe showing the most evident positive association (10). A recent systematic review and meta-analysis additionally reported a positive association between dietary AAA intake and hypertension risk; however, the available studies differed substantially in terms of population characteristics, dietary assessment methods, exposure definitions, and covariate adjustments (11). Our study has several important dimensions. Firstly, we evaluated the total AAA intake and individual components separately in a Chinese population with a distinct dietary pattern, characterized the dose–response relationships using restricted cubic spline models, and incorporated food source information to examine whether the observed associations differed across plant-, animal-, and mixed/other-derived AAA exposures. These analyses indicate that dietary AAAs should not be interpreted solely as total intake exposure, but rather as a group of metabolically related amino acids whose associations with hypertension may vary according to the component composition and food source context.

The divergent findings for Phe, Trp, and Tyr further support a component-specific interpretation of dietary AAAs. Although these amino acids share aromatic structures and are metabolically interconnected, they have distinct endogenous regulation, microbial metabolism, and downstream bioactive pathways (23–25). In light of the evidence linking Phe metabolism to gut microbial co-metabolism and cardiovascular risk, the more consistent positive association observed for Phe is biologically plausible. Gut microbial metabolism of Phe generates phenylacetic acid, which undergoes host conjugation to form phenylacetylglutamine (PAGln) (17). Experimental studies have indicated that PAGln modulates α2A-, α2B-, and β2-adrenergic receptor signaling and enhances platelet responsiveness (8, 26, 27). Trp-derived metabolites may further influence vascular function through distinct pathways. Quinolinic acid has been associated with markers of endothelial dysfunction in patients with chronic kidney disease (28), while serotonin can induce receptor-dependent vasoconstriction (29), and indoxyl sulfate can promote endothelial oxidative stress (30). More broadly, recent multi-omics and longitudinal studies have implicated gut microbiota-derived metabolites in blood pressure regulation and hypertension-related outcomes (31, 32). Isovalerylcarnitine is associated with salt sensitivity of blood pressure, as well as a lower risk of blood pressure progression and incident hypertension, while higher plasma PAGln concentrations are associated with an increased risk of major adverse cardiovascular events in high-risk patients with hypertension (32). The positive association with Trp may further reflect several bioactive pathways, because Trp can be metabolized through kynurenine, serotonin, and gut microbial indole pathways, which have been implicated in immune and inflammatory regulation, endothelial function, vascular tone, and blood pressure regulation (19–21). In contrast, the null findings for Tyr in the fully adjusted dose–response, cumulative average, and sensitivity analyses indicate that dietary Tyr may have a weaker independent relationship with hypertension risk in this population. One possible explanation for this is that dietary Tyr intake may not closely reflect Tyr availability or downstream pathway activity given its endogenous synthesis from Phe and its role in catecholamine synthesis (33, 34). Although the mean energy-adjusted Tyr intake differed between the lowest and highest quartiles (1.505 vs. 3.077 g/1,000 kcal), a small association cannot be ruled out, particularly given the uncertainty of food composition-based intake estimates. Together, these findings indicate that the association with total AAA may obscure the differences among its components, with more consistent positive associations observed for Phe and Trp than for Tyr.

### 4.2 Food-source context and dietary-pattern interpretation

One key contribution of our study is the source-specific evaluation of dietary AAAs, which allows these amino acids to be interpreted within their food matrix and dietary pattern contexts, rather than as isolated nutrients (13). In this Chinese cohort, plant-derived sources accounted for most of the total AAA and component intake, with cereals acting as the dominant contributor. This food source pattern should also be interpreted in the context of the ongoing nutritional transition in China. Longitudinal CHNS analyses have shown that a modern dietary pattern characterized by greater fruit and dairy intake was inversely associated with systolic blood pressure, while a meat-oriented pattern was associated with higher diastolic blood pressure and hypertension risk (35). More recent prospective evidence has further indicated that greater adherence to the Chinese Healthy Eating Index was associated with a lower risk of incident hypertension (36). Thus, plant-derived AAA intake in our study should not be equated with a healthy plant-rich dietary pattern dominated by vegetables, fruits, legumes, nuts, and whole grains. This distinction is important, because evidence from plant-based diet quality studies indicates that cardiometabolic associations differ substantially between healthy and less healthy plant foods, including refined grains and other lower-quality plant foods (37, 38). In the present study, plant-derived Phe and Trp showed modest positive associations, while plant-derived total AAA and Tyr showed no clear associations with hypertension. Because the total AAA combines components with potentially different association patterns, its aggregate estimate does not mirror each component estimate; however, differences in statistical significance alone do not establish whether these associations differ from one another. Although cereals were the largest contributors to plant-derived AAA intake, these exposures may further reflect broader differences in food choices and diet quality. Although Model 3 was adjusted for total energy, estimated sodium and potassium intake, and multiple sociodemographic and lifestyle factors, it did not account for total carbohydrate intake, dietary fiber, glycemic load, grain refinement, or a plant-based diet quality index. Additionally, sodium from discretionary salt and condiments consumed in cereal-based meals may have been incompletely captured. Therefore, residual confounding by these aspects of the broader dietary pattern is possible, and the observed associations for plant-derived Phe and Trp cannot be attributed to amino acids independent of their food sources. Further analyses distinguishing cereal-derived AAAs from other plant-derived AAA and incorporating markers of plant food quality, such as dietary fiber, may help to clarify these associations.

The inverse estimates of animal-derived AAA measures warrant cautious interpretation. In the mutually adjusted models, HRs per 1-SD increment ranged from 0.92 to 0.93. Similar inverse estimates were observed in fully adjusted single-source models (Supplementary Table S3), indicating that mutual adjustment for plant-derived intake alone is unlikely to explain this pattern. However, the crude single-source associations were positive and inverse after covariate adjustment, underscoring the sensitivity of these estimates to the model specification, the possibility of residual confounding by socioeconomic circumstances, and broader dietary patterns. Overall, we found no clear evidence of any interaction between plant- and animal-derived intakes (Supplementary Table S12), although this does not establish that their associations are independent. The inverse estimates should not be interpreted as evidence that animal-derived AAAs are protective, and our models did not estimate the effect of replacing a specified amount of plant-derived AAAs with equal amounts from animal sources. More broadly, Chinese cohort studies have associated a greater variety of protein sources consumed in appropriate amounts with a lower risk of incident hypertension and diabetes (39, 40). The animal-derived category also encompasses foods with heterogeneous cardiometabolic profiles, including red and processed meat, poultry, fish, eggs, dairy products, and offal; accordingly, associations may depend on the specific food source and replacement context (41, 42). These source-specific findings should be interpreted in the context of individual amino acids, food contributors, and dietary patterns. Further analyses distinguishing cereal-derived AAAs from other plant-derived AAA and incorporating markers of plant food quality, such as dietary fiber, may help to clarify these associations.

### 4.3 Strengths and limitations

This study has several strengths. First, it enrolled a large prospective cohort of Chinese adults with long-term follow-up and a substantial number of incident hypertension cases. Further, the longitudinal design of the database allowed dietary AAA intake to be assessed before disease onset, thereby reducing the likelihood of reverse causation. Second, dietary intake was assessed using 3 consecutive 24-h recalls, combined with household food weighing, while hypertension was ascertained using standardized blood pressure measurements and clinical information. Third, we evaluated Phe, Tyr, and Trp separately from the total AAA, examined dose–response patterns and food sources, and conducted subgroup and multiple sensitivity analyses. Fourth, mixed-effects Cox models accounted for household clustering.

Nevertheless, this study has some limitations. First, the dietary assessment was subject to measurement errors. Notably, AAA intake estimated from food composition tables may not fully capture the variations across foods, cooking methods, or survey periods, while food-based estimates do not include potential contributions from dietary supplements. Although 62.0% of the participants had repeated dietary assessments, dietary data were available only through 2011, whereas hypertension follow-up continued through 2015; subsequent dietary changes could therefore have introduced exposure misclassification. Second, despite the use of continuous and restricted cubic spline analyses to reduce reliance on exposure categories, estimates at the extreme tails of the intake distributions were less precise. Third, histories of myocardial infarction and stroke were based on self-reported physician diagnoses and may have been subject to recall errors and misclassification. Fourth, our data lacked circulating amino acid profiles and microbial metabolites, including phenylacetylglutamine, kynurenine pathway metabolites, and indole derivatives, thus limiting the mechanistic inference. Fifth, the food source categories reflect complex food matrices and broader dietary patterns. We did not formally evaluate the overall dietary patterns, such as DASH adherence or traditional Chinese dietary patterns, and the source-specific models did not include formal substitution analyses. Sixth, although a household-level random intercept accounted for within-household clustering, community-level clustering was not modeled separately. Residual within-community correlation may have affected the precision of estimates, while shared community characteristics may have contributed to residual confounding. Seventh, although Model 3 was adjusted for a range of potential confounders, a family history of hypertension was not included in the analysis. Residual confounders owing to familial susceptibility, broader dietary patterns, or other unmeasured factors therefore cannot be excluded. Thus, despite the temporal sequence established by the prospective follow-up, the observed associations warrant cautious interpretation of causality. Eighth, because cereals contributed substantially to the total AAA intake in this cohort (46.1%), the generalizability of the findings to populations with different staple food and protein source profiles remains uncertain. Further studies with detailed regional dietary information and measurements of circulating amino acids and microbial metabolites may help to clarify these findings.

## 5 Conclusion

Overall, this study found that higher dietary intakes of Phe and Trp were associated with a higher risk of incident hypertension among Chinese adults, while no clear association was observed for Tyr. The association for total AAA was modest and became weaker after adjusting for baseline province, indicating that regional differences may partly account for this finding. Associations also differed according to the food source.

## Data Availability

Publicly available datasets were analyzed in this study. This data can be found at: https://chns.cpc.unc.edu/.
