# Supplementary material for: Dietary aromatic amino acids, their food sources, and incident hypertension in Chinese adults: a prospective cohort study
Source: Front Nutr. 2026 Oct 22;13:1956118. doi: 10.3389/fnut.2026.1956118 (PMC13736745; doi:10.3389/fnut.2026.1956118)
Supplement: Supplementary file 1 [file Supplementary_file_1.docx]

**Supplementary Material**

**Dietary intake of aromatic amino acids and incident hypertension in Chinese adults: a prospective cohort study**

Ming Gong^1†^, Haiying Gao^2†^, Huiying Yu^3^, Wenlong Wu^1^,

Xue Gao^4^,Yingzhen Bu^1^,Xiangpeng Kong^1^**^*^** Xin Zhao^1^**^*^**

**Affiliations:**

^1^ Department of Cardiology, the Second Hospital of Dalian Medical University, No. 467 Zhongshan Road, Shahekou District, Dalian 116023, China

^2^ Department of General Medicine, the Second Hospital of Dalian Medical University, No. 467 Zhongshan Road, Shahekou District, Dalian 116023, China

^3^ Laboratory of Basic Medicine, General Hospital of Northern Theater Command, No. 83 Wenhua Road, Shenhe District, Shenyang 110016, China

^4^ The First Clinical College, Liaoning University of Traditional Chinese Medicine, No. 79 Chongshan East Road, Huanggu District, Shenyang 110847, China

† Ming Gong, Haiying Gao, and Xiangpeng Kong contributed equally to this work.

**^*^Corresponding author:**

**Xin Zhao**

Department of Cardiology, the Second Hospital of Dalian Medical University, No. 467 Zhongshan Road, Shahekou District, Dalian 116023, China

Email: zx81830@163.com

1. **Supplementary Table S1.** Baseline characteristics according to quartiles of total aromatic amino acid intake and its components
2. **Supplementary Table S2.** Associations of aromatic amino acid intake and its components with incident hypertension
3. **Supplementary Table S3.** Source-specific aromatic amino acid intake and risk of incident hypertension in single-source Cox models
4. **Supplementary Table S4.** Subgroup analyses of the associations between aromatic amino acid intake and incident hypertension
5. **Supplementary Table S5.** Sensitivity analysis using cumulative average aromatic amino acid intake
6. **Supplementary Table S6.** Sensitivity analyses for associations of aromatic amino acid intake with incident hypertension
7. **Supplementary Table S7.** Sensitivity analyses using alternative exposure categorizations
8. **Supplementary Table S8.** Time-stratified sensitivity analyses of aromatic amino acid intake and incident hypertension
9. **Supplementary Table S9**. Province-adjusted sensitivity analysis of dietary aromatic amino acid intake and incident hypertension
10. **Supplementary Table S10.** Missingness of baseline covariates and associations with dietary aromatic amino acid intake and incident hypertension
11. **1Supplementary Table S11.** Schoenfeld residual diagnostics of aromatic amino acid intake and incident hypertension (新增)
12. **Supplementary Table S12.** Interaction between plant- and animal-derived aromatic amino acid intakes and incident hypertension (新增)
13. **Supplementary Figure S1.** Correlation heatmap of total and source-specific aromatic amino acid intakes and total protein
14. **Supplementary Methods.**

**Supplementary Table S1. Baseline characteristics according to quartiles of total aromatic amino acid intake and its components**

**Panel A. Total aromatic amino acids (AAA)**

| **Variable** | **Overall** | **Q1** | **Q2** | **Q3** | **Q4** | **P value** |
| --- | --- | --- | --- | --- | --- | --- |
|  | n=10,087 | n=2,528 | n=2,517 | n=2,520 | n=2,522 |  |
| **Sociodemographic characteristics** | | | | | | |
| Age, y | 42.6±14.1 | 42.1±14.2 | 42.2±13.9 | 43.0±14.0 | 43.2±14.1 | 0.013 |
| Female, n (%) | 5474 (54.3) | 1296 (51.3) | 1322 (52.5) | 1410 (56.0) | 1446 (57.3) | <0.001 |
| Urban residence, n (%) | 4046 (40.1) | 683 (27.0) | 955 (37.9) | 1173 (46.5) | 1235 (49.0) | <0.001 |
| Baseline SBP, mmHg | 115.5±12.2 | 115.1±12.3 | 115.3±12.3 | 115.7±11.8 | 115.9±12.3 | 0.097 |
| Baseline DBP, mmHg | 75.3±8.5 | 75.0±8.7 | 75.3±8.5 | 75.5±8.3 | 75.4±8.6 | 0.072 |
| **Income level, n (%)** |  |  |  |  |  | <0.001 |
| Low | 2282 (27.6) | 860 (40.2) | 676 (31.7) | 425 (21.1) | 321 (16.2) |  |
| Medium | 3146 (38.1) | 909 (42.5) | 908 (42.6) | 733 (36.4) | 596 (30.1) |  |
| High | 2835 (34.3) | 369 (17.3) | 546 (25.6) | 857 (42.5) | 1063 (53.7) |  |
| **Education level, n (%)** |  |  |  |  |  | <0.001 |
| No formal education | 1044 (11.4) | 386 (17.2) | 287 (13.0) | 206 (8.9) | 165 (7.0) |  |
| ≤9 years | 5359 (58.7) | 1476 (65.9) | 1328 (60.0) | 1287 (55.7) | 1268 (53.6) |  |
| >9 years | 2728 (29.9) | 379 (16.9) | 599 (27.1) | 817 (35.4) | 933 (39.4) |  |
| **Physical activity level, n (%)** |  |  |  |  |  | <0.001 |
| Low | 2553 (25.4) | 758 (30.0) | 653 (26.0) | 620 (24.6) | 522 (20.7) |  |
| Moderate | 3281 (32.6) | 747 (29.6) | 764 (30.4) | 828 (32.9) | 942 (37.4) |  |
| High | 4236 (42.1) | 1019 (40.4) | 1094 (43.6) | 1071 (42.5) | 1052 (41.8) |  |
| **Lifestyle and adiposity** | | | | | | |
| Smoking, n (%) | 3094 (30.7) | 863 (34.2) | 791 (31.5) | 742 (29.5) | 698 (27.7) | <0.001 |
| Alcohol drinking, n (%) | 3475 (34.8) | 919 (36.8) | 881 (35.4) | 866 (34.6) | 809 (32.2) | 0.006 |
| BMI, kg/m² | 22.7±3.3 | 22.2±3.1 | 22.7±3.0 | 22.8±3.4 | 23.0±3.5 | <0.001 |
| Abdominal obesity, n (%) | 2046 (20.3) | 423 (16.7) | 480 (19.1) | 526 (20.9) | 617 (24.5) | <0.001 |
| **Medical history** | | | | | | |
| Diabetes, n (%) | 132 (1.3) | 20 (0.8) | 29 (1.2) | 34 (1.3) | 49 (1.9) | 0.003 |
| Stroke, n (%) | 31 (0.3) | 12 (0.5) | 4 (0.2) | 6 (0.2) | 9 (0.4) | 0.194 |
| Myocardial infarction, n (%) | 22 (0.2) | 6 (0.2) | 7 (0.3) | 6 (0.2) | 3 (0.1) | 0.649 |
| **Dietary intake** | | | | | | |
| Energy intake, kcal/d | 2136.0±625.8 | 2337.7±601.3 | 2268.2±590.1 | 2074.9±606.6 | 1863.1±594.0 | <0.001 |
| Carbohydrate, g/d | 310.0±119.6 | 376.2±115.7 | 335.7±109.0 | 287.1±104.9 | 240.9±103.1 | <0.001 |
| Protein, g/d | 67.2±22.8 | 66.6±19.8 | 68.8±21.7 | 67.3±23.4 | 66.2±25.9 | <0.001 |
| Fat, g/d | 67.8±35.2 | 60.2±33.3 | 70.2±34.8 | 71.5±35.9 | 69.5±35.8 | <0.001 |
| Sodium, g/d | 1.3±3.8 | 0.5±1.5 | 0.7±1.9 | 1.5±2.3 | 2.3±6.6 | <0.001 |
| Potassium, g/d | 2.2±3.3 | 0.9±2.0 | 1.3±2.3 | 2.7±3.2 | 4.1±4.2 | <0.001 |
| **Energy-adjusted amino acid intakes** | | | | | | |
| AAA, g/1000 kcal | 4.712±1.539 | 3.062±0.372 | 3.997±0.250 | 4.989±0.335 | 6.804±1.210 | <0.001 |
| Phe, g/1000 kcal | 1.843±0.648 | 1.182±0.200 | 1.615±0.231 | 1.987±0.311 | 2.589±0.649 | <0.001 |
| Trp, g/1000 kcal | 0.634±0.223 | 0.433±0.094 | 0.536±0.101 | 0.659±0.130 | 0.908±0.190 | <0.001 |
| Tyr, g/1000 kcal | 2.242±0.789 | 1.451±0.181 | 1.850±0.189 | 2.352±0.285 | 3.316±0.626 | <0.001 |

**Supplementary Table S1 (continued)**

**Panel B. Phenylalanine (Phe)**

| **Variable** | **Overall** | **Q1** | **Q2** | **Q3** | **Q4** | **P value** |
| --- | --- | --- | --- | --- | --- | --- |
|  | n=10,087 | n=2,522 | n=2,525 | n=2,518 | n=2,522 |  |
| **Sociodemographic characteristics** | | | | | | |
| Age, y | 42.6±14.1 | 41.9±14.2 | 42.1±13.8 | 43.0±14.0 | 43.5±14.2 | <0.001 |
| Female, n (%) | 5474 (54.3) | 1249 (49.5) | 1357 (53.7) | 1396 (55.4) | 1472 (58.4) | <0.001 |
| Urban residence, n (%) | 4046 (40.1) | 629 (24.9) | 887 (35.1) | 1262 (50.1) | 1268 (50.3) | <0.001 |
| Baseline SBP, mmHg | 115.5±12.2 | 115.0±12.4 | 115.0±12.2 | 116.1±12.1 | 116.1±12.2 | <0.001 |
| Baseline DBP, mmHg | 75.3±8.5 | 74.9±8.8 | 75.2±8.4 | 75.7±8.4 | 75.3±8.4 | 0.008 |
| **Income level, n (%)** |  |  |  |  |  | <0.001 |
| Low | 2282 (27.6) | 931 (42.8) | 705 (32.9) | 373 (18.7) | 273 (14.0) |  |
| Medium | 3146 (38.1) | 935 (43.0) | 948 (44.2) | 733 (36.7) | 530 (27.2) |  |
| High | 2835 (34.3) | 307 (14.1) | 493 (23.0) | 890 (44.6) | 1145 (58.8) |  |
| **Education level, n (%)** |  |  |  |  |  | <0.001 |
| No formal education | 1044 (11.4) | 394 (17.7) | 309 (13.9) | 196 (8.6) | 145 (6.0) |  |
| ≤9 years | 5359 (58.7) | 1487 (66.8) | 1355 (61.1) | 1232 (53.9) | 1285 (53.5) |  |
| >9 years | 2728 (29.9) | 346 (15.5) | 553 (24.9) | 856 (37.5) | 973 (40.5) |  |
| **Physical activity level, n (%)** |  |  |  |  |  | <0.001 |
| Low | 2553 (25.4) | 778 (30.9) | 664 (26.3) | 595 (23.6) | 516 (20.5) |  |
| Moderate | 3281 (32.6) | 734 (29.2) | 711 (28.2) | 861 (34.2) | 975 (38.8) |  |
| High | 4236 (42.1) | 1004 (39.9) | 1146 (45.5) | 1062 (42.2) | 1024 (40.7) |  |
| **Lifestyle and adiposity** | | | | | | |
| Smoking, n (%) | 3094 (30.7) | 883 (35.1) | 778 (30.9) | 755 (30.0) | 678 (26.9) | <0.001 |
| Alcohol drinking, n (%) | 3475 (34.8) | 944 (38.0) | 857 (34.4) | 889 (35.4) | 785 (31.2) | <0.001 |
| BMI, kg/m² | 22.7±3.3 | 22.1±3.0 | 22.6±3.0 | 22.9±3.4 | 23.1±3.6 | <0.001 |
| Abdominal obesity, n (%) | 2046 (20.3) | 390 (15.5) | 453 (17.9) | 553 (22.0) | 650 (25.8) | <0.001 |
| **Medical history** | | | | | | |
| Diabetes, n (%) | 132 (1.3) | 15 (0.6) | 31 (1.2) | 34 (1.4) | 52 (2.1) | <0.001 |
| Stroke, n (%) | 31 (0.3) | 9 (0.4) | 5 (0.2) | 11 (0.4) | 6 (0.2) | 0.953 |
| Myocardial infarction, n (%) | 22 (0.2) | 8 (0.3) | 4 (0.2) | 6 (0.2) | 4 (0.2) | 0.823 |
| **Dietary intake** | | | | | | |
| Energy intake, kcal/d | 2136.0±625.8 | 2370.8±595.3 | 2274.1±575.2 | 2058.5±604.1 | 1840.5±591.0 | <0.001 |
| Carbohydrate, g/d | 310.0±119.6 | 384.1±113.5 | 341.5±106.7 | 278.6±100.7 | 235.8±99.3 | <0.001 |
| Protein, g/d | 67.2±22.8 | 66.4±19.8 | 68.5±20.8 | 67.8±23.5 | 66.2±26.5 | <0.001 |
| Fat, g/d | 67.8±35.2 | 60.0±33.3 | 68.7±34.0 | 73.4±36.3 | 69.2±35.4 | <0.001 |
| Sodium, g/d | 1.3±3.8 | 0.4±1.3 | 0.6±1.6 | 1.6±2.5 | 2.5±6.7 | <0.001 |
| Potassium, g/d | 2.2±3.3 | 0.7±1.7 | 1.0±2.1 | 2.8±3.1 | 4.5±4.2 | <0.001 |
| **Energy-adjusted amino acid intakes** | | | | | | |
| AAA, g/1000 kcal | 4.710±1.539 | 3.113±0.436 | 4.041±0.442 | 4.968±0.532 | 6.750±1.253 | <0.001 |
| Phe, g/1000 kcal | 1.757±0.739 | 1.329±0.304 | 1.653±0.370 | 1.940±0.388 | 2.466±0.714 | <0.001 |
| Trp, g/1000 kcal | 0.638±0.236 | 0.446±0.082 | 0.573±0.099 | 0.693±0.106 | 0.838±0.191 | <0.001 |
| Tyr, g/1000 kcal | 2.231±0.800 | 1.439±0.159 | 1.836±0.125 | 2.358±0.206 | 3.291±0.592 | <0.001 |

**Supplementary Table S1 (continued)**

**Panel C. Tyrosine(Tyr)**

| **Variable** | **Overall** | **Q1** | **Q2** | **Q3** | **Q4** | **P value** |
| --- | --- | --- | --- | --- | --- | --- |
|  | n=10,087 | n=2,526 | n=2,520 | n=2,520 | n=2,521 |  |
| **Sociodemographic characteristics** | | | | | | |
| Age, y | 42.6±14.1 | 42.2±14.1 | 42.5±14.0 | 42.5±14.0 | 43.3±14.1 | 0.043 |
| Female, n (%) | 5474 (54.3) | 1321 (52.3) | 1323 (52.5) | 1396 (55.4) | 1434 (56.9) | 0.002 |
| Urban residence, n (%) | 4046 (40.1) | 746 (29.5) | 1053 (41.8) | 1158 (46.0) | 1089 (43.2) | <0.001 |
| Baseline SBP, mmHg | 115.5±12.2 | 115.4±12.3 | 115.4±12.2 | 115.9±12.0 | 115.5±12.4 | 0.410 |
| Baseline DBP, mmHg | 75.3±8.5 | 75.1±8.7 | 75.5±8.3 | 75.3±8.4 | 75.4±8.6 | 0.520 |
| **Income level, n (%)** |  |  |  |  |  | <0.001 |
| Low | 2282 (27.6) | 788 (37.4) | 583 (28.0) | 455 (22.6) | 456 (22.1) |  |
| Medium | 3146 (38.1) | 882 (41.9) | 828 (39.8) | 716 (35.6) | 720 (34.8) |  |
| High | 2835 (34.3) | 437 (20.7) | 670 (32.2) | 838 (41.7) | 890 (43.1) |  |
| **Education level, n (%)** |  |  |  |  |  | <0.001 |
| No formal education | 1044 (11.4) | 348 (13.9) | 257 (10.3) | 201 (8.0) | 238 (9.5) |  |
| ≤9 years | 5359 (58.7) | 1464 (58.5) | 1348 (53.9) | 1301 (52.0) | 1246 (49.9) |  |
| >9 years | 2728 (29.9) | 438 (17.5) | 664 (26.5) | 821 (32.8) | 805 (32.2) |  |
| **Physical activity level, n (%)** |  |  |  |  |  | <0.001 |
| Low | 2553 (25.4) | 712 (28.2) | 679 (27.0) | 618 (24.5) | 544 (21.6) |  |
| Moderate | 3281 (32.6) | 759 (30.1) | 808 (32.2) | 867 (34.4) | 847 (33.7) |  |
| High | 4236 (42.1) | 1051 (41.7) | 1025 (40.8) | 1034 (41.0) | 1126 (44.7) |  |
| **Lifestyle and adiposity** | | | | | | |
| Smoking, n (%) | 3094 (30.7) | 830 (32.9) | 786 (31.2) | 743 (29.5) | 735 (29.2) | 0.015 |
| Alcohol drinking, n (%) | 3475 (34.8) | 903 (36.2) | 893 (35.7) | 839 (33.5) | 840 (33.6) | 0.092 |
| BMI, kg/m² | 22.7±3.3 | 22.4±3.1 | 22.8±3.1 | 22.8±3.5 | 22.8±3.3 | <0.001 |
| Abdominal obesity, n (%) | 2046 (20.3) | 463 (18.3) | 494 (19.6) | 536 (21.3) | 553 (21.9) | 0.006 |
| **Medical history** | | | | | | |
| Diabetes, n (%) | 132 (1.3) | 24 (1.0) | 30 (1.2) | 31 (1.2) | 47 (1.9) | 0.030 |
| Stroke, n (%) | 31 (0.3) | 11 (0.4) | 7 (0.3) | 8 (0.3) | 5 (0.2) | 0.492 |
| Myocardial infarction, n (%) | 22 (0.2) | 7 (0.3) | 8 (0.3) | 4 (0.2) | 3 (0.1) | 0.379 |
| **Dietary intake** | | | | | | |
| Energy intake, kcal/d | 2136.0±625.8 | 2300.8±601.6 | 2229.4±611.3 | 2089.4±604.3 | 1924.2±619.1 | <0.001 |
| Carbohydrate, g/d | 310.0±119.6 | 362.8±114.5 | 325.6±111.6 | 293.7±109.8 | 257.9±116.6 | <0.001 |
| Protein, g/d | 67.2±22.8 | 66.4±20.0 | 68.8±22.2 | 67.8±23.4 | 65.9±25.3 | <0.001 |
| Fat, g/d | 67.8±35.2 | 62.2±33.8 | 70.5±35.3 | 70.2±35.0 | 68.5±36.2 | <0.001 |
| Sodium, g/d | 1.3±3.8 | 0.7±1.7 | 1.1±2.1 | 1.6±2.4 | 1.8±6.6 | <0.001 |
| Potassium, g/d | 2.2±3.3 | 1.2±2.3 | 1.9±2.8 | 2.9±3.3 | 2.9±4.3 | <0.001 |
| **Energy-adjusted amino acid intakes** | | | | | | |
| AAA, g/1000 kcal | 4.712±1.539 | 3.113±0.436 | 4.091±0.478 | 5.078±0.686 | 6.570±1.433 | <0.001 |
| Phe, g/1000 kcal | 1.843±0.648 | 1.154±0.158 | 1.575±0.108 | 1.957±0.120 | 2.687±0.595 | <0.001 |
| Trp, g/1000 kcal | 0.634±0.223 | 0.456±0.100 | 0.571±0.125 | 0.690±0.170 | 0.820±0.264 | <0.001 |
| Tyr, g/1000 kcal | 2.242±0.789 | 1.505±0.237 | 1.951±0.334 | 2.438±0.492 | 3.077±0.845 | <0.001 |

**Supplementary Table S1 (continued)**

**Panel D. Tryptophan (Trp)**

| **Variable** | **Overall** | **Q1** | **Q2** | **Q3** | **Q4** | **P value** |
| --- | --- | --- | --- | --- | --- | --- |
|  | n=10,087 | n=2,538 | n=2,512 | n=2,523 | n=2,514 |  |
| **Sociodemographic characteristics** | | | | | | |
| Age, y | 42.6±14.1 | 41.7±13.9 | 42.3±13.9 | 42.7±14.1 | 43.7±14.3 | <0.001 |
| Female, n (%) | 5474 (54.3) | 1299 (51.2) | 1316 (52.4) | 1407 (55.8) | 1452 (57.8) | <0.001 |
| Urban residence, n (%) | 4046 (40.1) | 546 (21.5) | 941 (37.5) | 1305 (51.7) | 1254 (49.9) | <0.001 |
| Baseline SBP, mmHg | 115.5±12.2 | 115.3±12.3 | 115.1±12.4 | 115.6±11.9 | 116.2±12.3 | 0.013 |
| Baseline DBP, mmHg | 75.3±8.5 | 75.2±8.9 | 75.1±8.5 | 75.5±8.1 | 75.4±8.5 | 0.412 |
| **Income level, n (%)** |  |  |  |  |  | <0.001 |
| Low | 2282 (27.6) | 997 (45.7) | 639 (30.3) | 367 (18.0) | 279 (14.4) |  |
| Medium | 3146 (38.1) | 889 (40.8) | 902 (42.7) | 794 (38.9) | 561 (29.1) |  |
| High | 2835 (34.3) | 294 (13.5) | 570 (27.0) | 880 (43.1) | 1091 (56.5) |  |
| **Education level, n (%)** |  |  |  |  |  | <0.001 |
| No formal education | 1044 (11.4) | 453 (18.0) | 260 (10.5) | 174 (6.9) | 157 (6.3) |  |
| ≤9 years | 5359 (58.7) | 1463 (58.2) | 1362 (54.9) | 1226 (48.9) | 1308 (52.2) |  |
| >9 years | 2728 (29.9) | 337 (13.4) | 603 (24.3) | 855 (34.1) | 933 (37.2) |  |
| **Physical activity level, n (%)** |  |  |  |  |  | <0.001 |
| Low | 2553 (25.4) | 794 (31.3) | 660 (26.4) | 569 (22.6) | 530 (21.1) |  |
| Moderate | 3281 (32.6) | 657 (25.9) | 791 (31.6) | 875 (34.7) | 958 (38.2) |  |
| High | 4236 (42.1) | 1085 (42.8) | 1053 (42.1) | 1075 (42.7) | 1023 (40.7) |  |
| **Lifestyle and adiposity** | | | | | | |
| Smoking, n (%) | 3094 (30.7) | 878 (34.7) | 814 (32.5) | 725 (28.8) | 677 (27.0) | <0.001 |
| Alcohol drinking, n (%) | 3475 (34.8) | 927 (37.0) | 899 (36.3) | 863 (34.4) | 786 (31.4) | <0.001 |
| BMI, kg/m² | 22.7±3.3 | 22.3±3.0 | 22.5±3.1 | 22.9±3.4 | 23.0±3.5 | <0.001 |
| Abdominal obesity, n (%) | 2046 (20.3) | 410 (16.2) | 442 (17.6) | 574 (22.8) | 620 (24.7) | <0.001 |
| **Medical history** | | | | | | |
| Diabetes, n (%) | 132 (1.3) | 19 (0.7) | 29 (1.2) | 32 (1.3) | 52 (2.1) | <0.001 |
| Stroke, n (%) | 31 (0.3) | 9 (0.4) | 7 (0.3) | 7 (0.3) | 8 (0.3) | 0.953 |
| Myocardial infarction, n (%) | 22 (0.2) | 6 (0.2) | 7 (0.3) | 5 (0.2) | 4 (0.2) | 0.823 |
| **Dietary intake** | | | | | | |
| Energy intake, kcal/d | 2136.0±625.8 | 2356.0±608.4 | 2220.7±580.2 | 2071.9±618.7 | 1893.8±599.0 | <0.001 |
| Carbohydrate, g/d | 310.0±119.6 | 393.3±118.7 | 329.1±101.5 | 272.5±96.1 | 244.6±102.8 | <0.001 |
| Protein, g/d | 67.2±22.8 | 66.2±20.4 | 66.4±21.6 | 69.3±24.8 | 66.9±24.1 | <0.001 |
| Fat, g/d | 67.8±35.2 | 54.8±30.2 | 69.0±34.0 | 76.7±36.9 | 70.9±35.7 | <0.001 |
| Sodium, g/d | 1.3±3.8 | 0.5±1.5 | 0.7±1.9 | 1.6±2.5 | 2.4±6.7 | <0.001 |
| Potassium, g/d | 2.2±3.3 | 0.9±2.0 | 1.3±2.3 | 2.7±3.2 | 4.1±4.2 | <0.001 |
| **Energy-adjusted amino acid intakes** | | | | | | |
| AAA, g/1000 kcal | 4.712±1.539 | 3.438±0.848 | 3.977±0.787 | 4.843±0.807 | 6.602±1.318 | <0.001 |
| Phe, g/1000 kcal | 1.843±0.648 | 1.483±0.580 | 1.618±0.517 | 1.866±0.460 | 2.409±0.602 | <0.001 |
| Trp, g/1000 kcal | 0.634±0.223 | 0.387±0.065 | 0.533±0.034 | 0.676±0.054 | 0.943±0.146 | <0.001 |
| Tyr, g/1000 kcal | 2.242±0.789 | 1.564±0.302 | 1.833±0.333 | 2.311±0.410 | 3.267±0.662 | <0.001 |

Note. Values are presented as means ± SDs for continuous variables and n (%) for categorical variables. Amino acid intakes were energy-adjusted and expressed as g/1000 kcal. Estimated sodium intake may be underestimated because discretionary salt and condiment use was not comprehensively captured. AAA, aromatic amino acids; Phe, phenylalanine; Tyr, tyrosine; Trp, tryptophan; SBP, systolic blood pressure; DBP, diastolic blood pressure; BMI, body mass index.

**Supplementary Table S2. Associations of aromatic amino acid intake and its components with incident hypertension**

| **Variable** | **Quartiles of intake** | | | | **P-trend** | **Per 1-SD increment** | **P for per SD** |
| --- | --- | --- | --- | --- | --- | --- | --- |
|  | **Q1** | **Q2** | **Q3** | **Q4** |  |  |  |
| **AAA (Total aromatic amino acids)** | | | | | | | |
| N | 2528 | 2517 | 2520 | 2522 |  | 10087 |  |
| Cases | 1050 | 972 | 804 | 705 |  | 3531 |  |
| Person-years | 29050.0 | 26605.0 | 21659.0 | 17839.0 |  | 95153.0 |  |
| Cases/1000 person-years | 36.1 | 36.5 | 37.1 | 39.5 |  | 37.1 |  |
| **Model estimates, HR (95% CI)** | | | | | | | |
| Crude model | 1.00 [Reference] | 1.08 [0.97, 1.19] | 1.24 [1.12, 1.39] | 1.60 [1.42, 1.79] | <0.001 | 1.17 [1.13, 1.22] | <0.001 |
| Model 1 | 1.00 [Reference] | 1.04 [0.94, 1.16] | 1.17 [1.04, 1.30] | 1.39 [1.24, 1.56] | <0.001 | 1.11 [1.06, 1.15] | <0.001 |
| Model 2 | 1.00 [Reference] | 1.01 [0.91, 1.11] | 1.17 [1.05, 1.30] | 1.32 [1.17, 1.48] | <0.001 | 1.10 [1.05, 1.14] | <0.001 |
| **Model 3** | **1.00 [Reference]** | **1.00 [0.91, 1.11]** | **1.12 [1.00, 1.24]** | **1.16 [1.03, 1.31]** | **0.006** | **1.05 [1.00, 1.09]** | **0.039** |
| **Phe (Phenylalanine)** | | | | | | | |
| N | 2522 | 2525 | 2518 | 2522 |  | 10087 |  |
| Cases | 1080 | 1006 | 786 | 659 |  | 3531 |  |
| Person-years | 30012.0 | 27905.0 | 20882.0 | 16354.0 |  | 95153.0 |  |
| Cases/1000 person-years | 36.0 | 36.1 | 37.6 | 40.3 |  | 37.1 |  |
| **Model estimates, HR (95% CI)** | | | | | | | |
| Crude model | 1.00 [Reference] | 1.05 [0.95, 1.16] | 1.36 [1.22, 1.52] | 1.85 [1.64, 2.07] | <0.001 | 1.26 [1.21, 1.32] | <0.001 |
| Model 1 | 1.00 [Reference] | 1.02 [0.92, 1.13] | 1.26 [1.13, 1.41] | 1.57 [1.39, 1.77] | <0.001 | 1.18 [1.13, 1.23] | <0.001 |
| Model 2 | 1.00 [Reference] | 1.00 [0.90, 1.10] | 1.21 [1.09, 1.36] | 1.48 [1.31, 1.68] | <0.001 | 1.15 [1.10, 1.20] | <0.001 |
| **Model 3** | **1.00 [Reference]** | **0.99 [0.90, 1.10]** | **1.15 [1.03, 1.28]** | **1.26 [1.11, 1.44]** | **<0.001** | **1.08 [1.04, 1.14]** | **<0.001** |
| **Tyr (Tyrosine)** | | | | | | | |
| N | 2526 | 2520 | 2520 | 2521 |  | 10087 |  |
| Cases | 1002 | 912 | 759 | 858 |  | 3531 |  |
| Person-years | 27705.0 | 24529.0 | 21257.0 | 21662.0 |  | 95153.0 |  |
| Cases/1000 person-years | 36.2 | 37.2 | 35.7 | 39.6 |  | 37.1 |  |
| **Model estimates, HR (95% CI)** | | | | | | | |
| Crude model | 1.00 [Reference] | 1.11 [1.00, 1.24] | 1.15 [1.03, 1.29] | 1.26 [1.13, 1.41] | <0.001 | 1.05 [1.02, 1.09] | 0.005 |
| Model 1 | 1.00 [Reference] | 1.06 [0.96, 1.18] | 1.09 [0.98, 1.22] | 1.16 [1.04, 1.30] | 0.006 | 1.03 [0.99, 1.06] | 0.178 |
| Model 2 | 1.00 [Reference] | 1.04 [0.94, 1.15] | 1.06 [0.95, 1.18] | 1.16 [1.04, 1.29] | 0.007 | 1.03 [0.99, 1.07] | 0.134 |
| **Model 3** | **1.00 [Reference]** | **1.04 [0.94, 1.15]** | **1.02 [0.91, 1.13]** | **1.09 [0.98, 1.21]** | **0.168** | **1.00 [0.97, 1.04]** | **0.871** |
| **Trp (Tryptophan)** | | | | | | | |
| N | 2538 | 2512 | 2523 | 2514 |  | 10087 |  |
| Cases | 1119 | 977 | 766 | 669 |  | 3531 |  |
| Person-years | 30384.0 | 26519.0 | 21551.0 | 16699.0 |  | 95153.0 |  |
| Cases/1000 person-years | 36.8 | 36.8 | 35.5 | 40.1 |  | 37.1 |  |
| **Model estimates, HR (95% CI)** | | | | | | | |
| Crude model | 1.00 [Reference] | 1.12 [1.01, 1.24] | 1.24 [1.12, 1.38] | 1.81 [1.62, 2.03] | <0.001 | 1.23 [1.18, 1.28] | <0.001 |
| Model 1 | 1.00 [Reference] | 1.03 [0.93, 1.14] | 1.08 [0.96, 1.21] | 1.48 [1.31, 1.67] | <0.001 | 1.13 [1.08, 1.18] | <0.001 |
| Model 2 | 1.00 [Reference] | 1.08 [0.97, 1.19] | 1.11 [0.99, 1.24] | 1.48 [1.31, 1.67] | <0.001 | 1.13 [1.09, 1.19] | <0.001 |
| **Model 3** | **1.00 [Reference]** | **1.06 [0.95, 1.17]** | **1.06 [0.95, 1.18]** | **1.29 [1.14, 1.46]** | **<0.001** | **1.08 [1.03, 1.13]** | **0.001** |

Note: Values are HRs (95% CIs) unless otherwise indicated. Q1 was the reference group. Per 1-SD increment indicates the HR associated with a 1-standard-deviation increase in the corresponding exposure. P-trend was calculated by assigning participants the median intake value of their corresponding quartile and modeling this value as a continuous variable. P for per SD was calculated for the continuous standardized exposure. The crude model was unadjusted. Model 1 was adjusted for age, sex, income level, education level, and urban residence. Model 2 was further adjusted for diabetes, myocardial infarction, stroke, smoking, alcohol drinking, baseline systolic blood pressure, BMI category, abdominal obesity, and physical activity level. Model 3 was further adjusted for total energy intake, sodium intake, and potassium intake. Mixed-effects Cox proportional hazards models included household identifier as a random intercept. Missing covariates were handled using multiple imputation by chained equations. AAA, aromatic amino acids; Phe, phenylalanine; Tyr, tyrosine; Trp, tryptophan; HR, hazard ratio; CI, confidence interval; BMI, body mass index.

**Supplementary Table S3. Source-specific aromatic amino acid intake and risk of incident hypertension in single-source Cox models**

| **Amino acid** | **Source** | **Model** | **HR (95% CI)** | **P value** |
| --- | --- | --- | --- | --- |
| AAA | Plant-derived | Crude model | 1.03 (0.99, 1.07) | 0.159 |
| AAA | Plant-derived | Model 1 | 1.07 (1.03, 1.11) | <0.001 |
| AAA | Plant-derived | Model 2 | 1.07 (1.03, 1.11) | <0.001 |
| AAA | Plant-derived | Model 3 | 1.04 (1.00, 1.08) | 0.062 |
| AAA | Animal-derived | Crude model | 1.10 (1.06, 1.13) | <0.001 |
| AAA | Animal-derived | Model 1 | 1.00 (0.95, 1.04) | 0.845 |
| AAA | Animal-derived | Model 2 | 0.97 (0.93, 1.02) | 0.254 |
| AAA | Animal-derived | Model 3 | 0.92 (0.87, 0.97) | <0.001 |
| AAA | Mixed/other-derived | Crude model | 1.10 (1.06, 1.14) | <0.001 |
| AAA | Mixed/other-derived | Model 1 | 1.06 (1.02, 1.10) | 0.005 |
| AAA | Mixed/other-derived | Model 2 | 1.04 (0.99, 1.08) | 0.101 |
| AAA | Mixed/other-derived | Model 3 | 0.89 (0.84, 0.94) | <0.001 |
| Phe | Plant-derived | Crude model | 1.10 (1.06, 1.15) | <0.001 |
| Phe | Plant-derived | Model 1 | 1.13 (1.08, 1.17) | <0.001 |
| Phe | Plant-derived | Model 2 | 1.11 (1.07, 1.16) | <0.001 |
| Phe | Plant-derived | Model 3 | 1.07 (1.03, 1.11) | 0.002 |
| Phe | Animal-derived | Crude model | 1.10 (1.07, 1.13) | <0.001 |
| Phe | Animal-derived | Model 1 | 1.00 (0.96, 1.05) | 0.900 |
| Phe | Animal-derived | Model 2 | 0.98 (0.93, 1.03) | 0.375 |
| Phe | Animal-derived | Model 3 | 0.92 (0.87, 0.97) | 0.001 |
| Phe | Mixed/other-derived | Crude model | 1.10 (1.07, 1.14) | <0.001 |
| Phe | Mixed/other-derived | Model 1 | 1.06 (1.02, 1.10) | 0.004 |
| Phe | Mixed/other-derived | Model 2 | 1.04 (1.00, 1.09) | 0.077 |
| Phe | Mixed/other-derived | Model 3 | 0.88 (0.83, 0.94) | <0.001 |
| Tyr | Plant-derived | Crude model | 0.94 (0.91, 0.98) | 0.003 |
| Tyr | Plant-derived | Model 1 | 0.99 (0.96, 1.03) | 0.773 |
| Tyr | Plant-derived | Model 2 | 1.01 (0.97, 1.04) | 0.742 |
| Tyr | Plant-derived | Model 3 | 1.00 (0.96, 1.03) | 0.831 |
| Tyr | Animal-derived | Crude model | 1.10 (1.07, 1.14) | <0.001 |
| Tyr | Animal-derived | Model 1 | 1.00 (0.96, 1.05) | 0.986 |
| Tyr | Animal-derived | Model 2 | 0.98 (0.93, 1.02) | 0.332 |
| Tyr | Animal-derived | Model 3 | 0.92 (0.87, 0.97) | 0.001 |
| Tyr | Mixed/other-derived | Crude model | 1.10 (1.06, 1.14) | <0.001 |
| Tyr | Mixed/other-derived | Model 1 | 1.06 (1.02, 1.10) | 0.006 |
| Tyr | Mixed/other-derived | Model 2 | 1.04 (0.99, 1.08) | 0.122 |
| Tyr | Mixed/other-derived | Model 3 | 0.90 (0.85, 0.95) | <0.001 |
| Trp | Plant-derived | Crude model | 1.12 (1.08, 1.17) | <0.001 |
| Trp | Plant-derived | Model 1 | 1.14 (1.09, 1.18) | <0.001 |
| Trp | Plant-derived | Model 2 | 1.14 (1.09, 1.19) | <0.001 |
| Trp | Plant-derived | Model 3 | 1.09 (1.04, 1.14) | <0.001 |
| Trp | Animal-derived | Crude model | 1.07 (1.04, 1.11) | <0.001 |
| Trp | Animal-derived | Model 1 | 0.96 (0.92, 1.01) | 0.084 |
| Trp | Animal-derived | Model 2 | 0.95 (0.90, 0.99) | 0.021 |
| Trp | Animal-derived | Model 3 | 0.92 (0.88, 0.96) | <0.001 |
| Trp | Mixed/other-derived | Crude model | 1.08 (1.04, 1.12) | <0.001 |
| Trp | Mixed/other-derived | Model 1 | 1.05 (1.00, 1.09) | 0.030 |
| Trp | Mixed/other-derived | Model 2 | 1.02 (0.98, 1.07) | 0.377 |
| Trp | Mixed/other-derived | Model 3 | 0.92 (0.87, 0.96) | <0.001 |

Note. Values are HRs and 95% CIs per 1-SD increment in source-specific amino acid intake. Each source-specific exposure was modeled separately. All analyses included 10,087 participants and 3,531 incident hypertension cases; estimates were pooled across 20 imputed datasets using Rubin’s rules. Model 1 adjusted for age, sex, urban residence, income level, and education level. Model 2 further adjusted for history of diabetes, myocardial infarction, stroke, smoking status, alcohol drinking, baseline systolic blood pressure, BMI category, abdominal obesity, and physical activity level. Model 3 further adjusted for total energy intake, sodium intake, and potassium intake. AAA, aromatic amino acids; Phe, phenylalanine; Tyr, tyrosine; Trp, tryptophan; HR, hazard ratio; CI, confidence interval; BMI, body mass index.

**Supplementary Table S4. Subgroup analyses of the associations between aromatic amino acid intake and incident hypertension**

| **Subgroup** | **Category** | **N** | **Cases** | **HR (95% CI)** | **P value** | **P for interaction** |
| --- | --- | --- | --- | --- | --- | --- |
| **AAA (total aromatic amino acids)** | | | | | | |
| **Age** | <60 | 8747 | 2827 | 1.06 (1.01, 1.11) | 0.014 | 0.077 |
|  | ≥60 | 1340 | 704 | 1.02 (0.93, 1.11) | 0.713 |  |
| **Sex** | Male | 4613 | 1825 | 1.00 (0.94, 1.07) | 0.909 | 0.010 |
|  | Female | 5474 | 1706 | 1.07 (1.01, 1.13) | 0.022 |  |
| **Residence** | Rural | 6041 | 2307 | 1.09 (1.04, 1.15) | <0.001 | <0.001 |
|  | Urban | 4046 | 1224 | 0.97 (0.90, 1.04) | 0.386 |  |
| **Income** | Low | 2728 | 1126 | 1.05 (0.97, 1.14) | 0.231 | 0.373 |
|  | Medium/high | 7360 | 2406 | 1.04 (0.99, 1.10) | 0.117 |  |
| **Education** | ≤9 years | 6462 | 2508 | 1.06 (1.01, 1.12) | 0.014 | <0.001 |
|  | >9 years | 3625 | 1023 | 0.96 (0.89, 1.05) | 0.392 |  |
| **Smoking status** | Never | 6989 | 2268 | 1.05 (1.00, 1.10) | 0.056 | 0.238 |
|  | Ever | 3098 | 1263 | 1.00 (0.94, 1.08) | 0.909 |  |
| **Alcohol drinking** | Never | 6589 | 2148 | 1.06 (1.01, 1.12) | 0.021 | 0.068 |
|  | Ever | 3498 | 1383 | 1.02 (0.95, 1.09) | 0.623 |  |
| **Overweight/obesity** | No | 7021 | 2156 | 1.06 (1.01, 1.12) | 0.026 | 0.340 |
|  | Yes | 3066 | 1375 | 1.01 (0.95, 1.08) | 0.666 |  |
| **Abdominal obesity** | No | 8041 | 2585 | 1.07 (1.01, 1.12) | 0.011 | 0.252 |
|  | Yes | 2046 | 946 | 0.99 (0.92, 1.07) | 0.878 |  |
| **Physical activity** | Low | 3364 | 1399 | 1.00 (0.93, 1.07) | 0.939 | 0.211 |
|  | Moderate/high | 6723 | 2132 | 1.06 (1.01, 1.11) | 0.024 |  |
| **Phe (phenylalanine)** | | | | | | |
| **Age** | <60 | 8747 | 2827 | 1.10 (1.04, 1.16) | <0.001 | 0.068 |
|  | ≥60 | 1340 | 704 | 1.05 (0.96, 1.16) | 0.285 |  |
| **Sex** | Male | 4613 | 1825 | 1.04 (0.97, 1.11) | 0.242 | 0.010 |
|  | Female | 5474 | 1706 | 1.09 (1.03, 1.16) | 0.003 |  |
| **Residence** | Rural | 6041 | 2307 | 1.14 (1.08, 1.21) | <0.001 | <0.001 |
|  | Urban | 4046 | 1224 | 1.00 (0.92, 1.08) | 0.941 |  |
| **Income** | Low | 2728 | 1126 | 1.06 (0.96, 1.17) | 0.265 | 0.536 |
|  | Medium/high | 7360 | 2406 | 1.09 (1.03, 1.15) | 0.002 |  |
| **Education** | ≤9 years | 6462 | 2508 | 1.10 (1.04, 1.16) | <0.001 | <0.001 |
|  | >9 years | 3625 | 1023 | 1.00 (0.92, 1.09) | 0.968 |  |
| **Smoking status** | Never | 6989 | 2268 | 1.07 (1.02, 1.13) | 0.010 | 0.250 |
|  | Ever | 3098 | 1263 | 1.04 (0.96, 1.12) | 0.300 |  |
| **Alcohol drinking** | Never | 6589 | 2148 | 1.10 (1.04, 1.16) | 0.002 | 0.075 |
|  | Ever | 3498 | 1383 | 1.05 (0.98, 1.14) | 0.157 |  |
| **Overweight/obesity** | No | 7021 | 2156 | 1.10 (1.04, 1.17) | 0.001 | 0.322 |
|  | Yes | 3066 | 1375 | 1.04 (0.97, 1.11) | 0.286 |  |
| **Abdominal obesity** | No | 8041 | 2585 | 1.10 (1.04, 1.17) | <0.001 | 0.349 |
|  | Yes | 2046 | 946 | 1.02 (0.94, 1.11) | 0.581 |  |
| **Physical activity** | Low | 3364 | 1399 | 1.02 (0.94, 1.10) | 0.692 | 0.303 |
|  | Moderate/high | 6723 | 2132 | 1.09 (1.03, 1.15) | 0.002 |  |
| **Tyr (tyrosine)** | | | | | | |
| **Age** | <60 | 8747 | 2827 | 1.02 (0.97, 1.06) | 0.481 | 0.288 |
|  | ≥60 | 1340 | 704 | 1.00 (0.91, 1.08) | 0.912 |  |
| **Sex** | Male | 4613 | 1825 | 0.97 (0.91, 1.02) | 0.202 | 0.012 |
|  | Female | 5474 | 1706 | 1.04 (0.99, 1.09) | 0.138 |  |
| **Residence** | Rural | 6041 | 2307 | 1.03 (0.99, 1.08) | 0.124 | <0.001 |
|  | Urban | 4046 | 1224 | 0.95 (0.88, 1.02) | 0.134 |  |
| **Income** | Low | 2728 | 1126 | 1.04 (0.97, 1.10) | 0.268 | 0.207 |
|  | Medium/high | 7360 | 2406 | 0.99 (0.95, 1.04) | 0.707 |  |
| **Education** | ≤9 years | 6462 | 2508 | 1.02 (0.98, 1.07) | 0.254 | <0.001 |
|  | >9 years | 3625 | 1023 | 0.93 (0.86, 1.01) | 0.070 |  |
| **Smoking status** | Never | 6989 | 2268 | 1.02 (0.98, 1.07) | 0.303 | 0.153 |
|  | Ever | 3098 | 1263 | 0.96 (0.91, 1.03) | 0.264 |  |
| **Alcohol drinking** | Never | 6589 | 2148 | 1.02 (0.98, 1.07) | 0.310 | 0.103 |
|  | Ever | 3498 | 1383 | 0.98 (0.93, 1.04) | 0.554 |  |
| **Overweight/obesity** | No | 7021 | 2156 | 1.02 (0.98, 1.07) | 0.332 | 0.282 |
|  | Yes | 3066 | 1375 | 0.99 (0.93, 1.05) | 0.636 |  |
| **Abdominal obesity** | No | 8041 | 2585 | 1.03 (0.98, 1.07) | 0.243 | 0.124 |
|  | Yes | 2046 | 946 | 0.96 (0.89, 1.03) | 0.260 |  |
| **Physical activity** | Low | 3364 | 1399 | 0.99 (0.93, 1.05) | 0.640 | 0.289 |
|  | Moderate/high | 6723 | 2132 | 1.02 (0.98, 1.07) | 0.367 |  |
| **Trp (tryptophan)** | | | | | | |
| **Age** | <60 | 8747 | 2827 | 1.09 (1.04, 1.15) | <0.001 | 0.044 |
|  | ≥60 | 1340 | 704 | 1.03 (0.94, 1.13) | 0.553 |  |
| **Sex** | Male | 4613 | 1825 | 1.05 (0.99, 1.12) | 0.111 | 0.154 |
|  | Female | 5474 | 1706 | 1.06 (1.00, 1.13) | 0.050 |  |
| **Residence** | Rural | 6041 | 2307 | 1.14 (1.07, 1.20) | <0.001 | <0.001 |
|  | Urban | 4046 | 1224 | 0.97 (0.90, 1.05) | 0.483 |  |
| **Income** | Low | 2728 | 1126 | 1.04 (0.94, 1.15) | 0.457 | 0.679 |
|  | Medium/high | 7360 | 2406 | 1.08 (1.02, 1.14) | 0.006 |  |
| **Education** | ≤9 years | 6462 | 2508 | 1.08 (1.02, 1.14) | 0.005 | <0.001 |
|  | >9 years | 3625 | 1023 | 1.01 (0.93, 1.10) | 0.821 |  |
| **Smoking status** | Never | 6989 | 2268 | 1.06 (1.00, 1.12) | 0.043 | 0.814 |
|  | Ever | 3098 | 1263 | 1.04 (0.96, 1.12) | 0.351 |  |
| **Alcohol drinking** | Never | 6589 | 2148 | 1.09 (1.03, 1.15) | 0.002 | 0.062 |
|  | Ever | 3498 | 1383 | 1.03 (0.95, 1.11) | 0.478 |  |
| **Overweight/obesity** | No | 7021 | 2156 | 1.06 (0.99, 1.12) | 0.079 | 0.696 |
|  | Yes | 3066 | 1375 | 1.07 (1.00, 1.14) | 0.051 |  |
| **Abdominal obesity** | No | 8041 | 2585 | 1.06 (1.01, 1.12) | 0.028 | 0.554 |
|  | Yes | 2046 | 946 | 1.08 (1.00, 1.17) | 0.065 |  |
| **Physical activity** | Low | 3364 | 1399 | 1.01 (0.93, 1.10) | 0.818 | 0.253 |
|  | Moderate/high | 6723 | 2132 | 1.08 (1.02, 1.14) | 0.007 |  |

Note. Values are HRs (95% CIs) for incident hypertension per 1-SD increment in energy-adjusted amino acid intake. Models were adjusted for age, sex, residence, income, education, history of diabetes, myocardial infarction, stroke, smoking status, alcohol drinking, baseline systolic blood pressure, BMI category, abdominal obesity, physical activity, total energy intake, sodium intake, and potassium intake, with household identifier included as a random intercept, except for the corresponding stratification variable. Estimates were pooled across 20 imputed datasets using Rubin’s rules. P-interaction values were obtained from models including cross-product terms between each amino acid exposure and the corresponding subgroup variable. AAA, aromatic amino acids; Phe, phenylalanine; Tyr, tyrosine; Trp, tryptophan; HR, hazard ratio; CI, confidence interval; BMI, body mass index.

**Supplementary Table S5. Sensitivity analysis using cumulative average intakes of aromatic amino acids and their components**

| Exposure | No. of participants | Cases | Observation intervals | HR (95% CI) | P value |
| --- | --- | --- | --- | --- | --- |
| AAA (total aromatic amino acids) | 10,087 | 3,531 | 28,309 | 1.04 (1.00, 1.08) | 0.059 |
| Phe (phenylalanine) | 10,087 | 3,531 | 28,309 | 1.07 (1.03, 1.11) | 0.001 |
| Tyr (tyrosine) | 10,087 | 3,531 | 28,309 | 1.00 (0.96, 1.04) | 0.914 |
| Trp (tryptophan) | 10,087 | 3,531 | 28,309 | 1.06 (1.02, 1.10) | 0.006 |

Note. Values are HRs (95% CIs) for incident hypertension per 1-SD increment in cumulatively averaged amino acid intake. Cumulative average intakes were calculated from repeated dietary assessments up to the beginning of each risk interval and used to predict incident hypertension during the subsequent interval. Models were adjusted for age, sex, income level, education level, urban residence, history of diabetes, myocardial infarction, stroke, smoking status, alcohol drinking, baseline systolic blood pressure, BMI category, abdominal obesity, physical activity level, total energy intake, sodium intake, and potassium intake, with household identifier included as a random intercept. Missing baseline covariates were handled using multiple imputation. AAA, aromatic amino acids; Phe, phenylalanine; Tyr, tyrosine; Trp, tryptophan; HR, hazard ratio; CI, confidence interval; BMI, body mass index*.*

**Supplementary Table S6. Sensitivity analyses for associations of aromatic amino acid intake with incident hypertension**

| **Sensitivity analysis** | **No. of participants** | **Cases** | **HR (95% CI)** | **P value** |
| --- | --- | --- | --- | --- |
| **AAA (Total aromatic amino acids)** | | | | |
| Complete-case analysis | 8072 | 2895 | 1.05 (1.01, 1.10) | 0.030 |
| Excluding events within first 2 years | 9988 | 3432 | 1.05 (1.01, 1.10) | 0.020 |
| Excluding baseline diabetes, myocardial infarction, or stroke | 9909 | 3456 | 1.05 (1.00, 1.09) | 0.040 |
| Further adjusted for total protein intake | 10087 | 3531 | 1.08 (1.03, 1.13) | 0.001 |
| **Phe (Phenylalanine)** | | | | |
| Complete-case analysis | 8072 | 2895 | 1.09 (1.03, 1.14) | 0.001 |
| Excluding events within first 2 years | 9988 | 3432 | 1.09 (1.04, 1.14) | <0.001 |
| Excluding baseline diabetes, myocardial infarction, or stroke | 9909 | 3456 | 1.09 (1.04, 1.14) | <0.001 |
| Further adjusted for total protein intake | 10087 | 3531 | 1.13 (1.07, 1.19) | <0.001 |
| **Tyr (Tyrosine)** | | | | |
| Complete-case analysis | 8072 | 2895 | 1.01 (0.97, 1.06) | 0.511 |
| Excluding events within first 2 years | 9988 | 3432 | 1.01 (0.98, 1.05) | 0.528 |
| Excluding baseline diabetes, myocardial infarction, or stroke | 9909 | 3456 | 1.00 (0.97, 1.04) | 0.864 |
| Further adjusted for total protein intake | 10087 | 3531 | 1.02 (0.98, 1.06) | 0.359 |
| **Trp (Tryptophan)** | | | | |
| Complete-case analysis | 8072 | 2895 | 1.08 (1.03, 1.14) | 0.002 |
| Excluding events within first 2 years | 9988 | 3432 | 1.08 (1.03, 1.13) | 0.002 |
| Excluding baseline diabetes, myocardial infarction, or stroke | 9909 | 3456 | 1.08 (1.03, 1.13) | 0.001 |
| Further adjusted for total protein intake | 10087 | 3531 | 1.12 (1.07, 1.18) | <0.001 |

Note. Values are HRs (95% CIs) for incident hypertension per 1-SD increment in energy-adjusted amino acid intake. Sensitivity analyses included complete-case analysis, exclusion of participants who developed hypertension within the first 2 years of follow-up, exclusion of participants with baseline diabetes, myocardial infarction, or stroke, and additional adjustment for total protein intake. Models were adjusted for age, sex, urban residence, income level, education level, history of diabetes, myocardial infarction, stroke, smoking status, alcohol drinking, baseline systolic blood pressure, BMI category, abdominal obesity, physical activity level, total energy intake, sodium intake, and potassium intake, with household identifier included as a random intercept. Missing covariates were handled using multiple imputation by chained equations, and estimates were pooled across 20 imputed datasets using Rubin’s rules, except for the complete-case analysis. AAA, aromatic amino acids; Phe, phenylalanine; Tyr, tyrosine; Trp, tryptophan; HR, hazard ratio; CI, confidence interval; BMI, body mass index.

**Supplementary Table S7. Sensitivity analyses using alternative exposure categorizations**

**Panel A. Tertile analysis**

| **Exposure** | **T1** | **T2** | **T3** | **P-trend** |
| --- | --- | --- | --- | --- |
| AAA | 1.00 [Reference] | 1.03 (0.94, 1.12) | 1.19 (1.07, 1.32) | 0.002 |
| Phe | 1.00 [Reference] | 1.06 (0.97, 1.16) | 1.24 (1.11, 1.39) | <0.001 |
| Tyr | 1.00 [Reference] | 1.00 (0.91, 1.09) | 1.05 (0.95, 1.15) | 0.381 |
| Trp | 1.00 [Reference] | 1.05 (0.95, 1.14) | 1.24 (1.11, 1.38) | <0.001 |

**Panel B. Quintile analysis**

| **Exposure** | **Q1** | **Q2** | **Q3** | **Q4** | **Q5** | **P-trend** |
| --- | --- | --- | --- | --- | --- | --- |
| AAA | 1.00 [Reference] | 0.94 (0.84, 1.05) | 1.04 (0.93, 1.17) | 1.18 (1.04, 1.34) | 1.13 (0.99, 1.29) | 0.003 |
| Phe | 1.00 [Reference] | 0.97 (0.87, 1.08) | 1.13 (1.01, 1.27) | 1.22 (1.07, 1.38) | 1.27 (1.10, 1.46) | <0.001 |
| Tyr | 1.00 [Reference] | 1.00 (0.89, 1.12) | 1.03 (0.92, 1.16) | 1.06 (0.94, 1.20) | 1.03 (0.91, 1.16) | 0.394 |
| Trp | 1.00 [Reference] | 1.01 (0.90, 1.12) | 1.05 (0.94, 1.18) | 1.13 (0.99, 1.28) | 1.29 (1.12, 1.48) | <0.001 |

Note. Values are HRs and 95% CIs for incident hypertension across tertiles or quintiles of energy-adjusted amino acid intake. T1 and Q1 were used as the reference groups. P-trend was calculated by modeling tertile or quintile categories as ordinal variables. All analyses included 10,087 participants and 3,531 incident hypertension cases. Models were adjusted for age, sex, urban residence, income level, education level, history of diabetes, myocardial infarction, stroke, smoking status, alcohol drinking, baseline systolic blood pressure, BMI category, abdominal obesity, physical activity level, total energy intake, sodium intake, and potassium intake, with household identifier included as a random intercept. Missing covariates were handled using multiple imputation by chained equations, and estimates were pooled across 20 imputed datasets using Rubin's rules. AAA, aromatic amino acids; Phe, phenylalanine; Tyr, tyrosine; Trp, tryptophan; HR, hazard ratio; CI, confidence interval; BMI, body mass index.

**Supplementary Table S8. Time-stratified sensitivity analyses of aromatic amino acid intake and incident hypertension**

| **Exposure** | **Follow-up period** | **Per 1-SD increment**  **HR (95% CI)** | **P value** | **Q4 vs Q1**  **HR (95% CI)** | **P value** |
| --- | --- | --- | --- | --- | --- |
| **AAA** | ≤8 years | 1.03 (0.97, 1.10) | 0.346 | 1.12 (0.93, 1.35) | 0.213 |
|  | >8 years | 1.03 (0.97, 1.08) | 0.312 | 1.12 (0.96, 1.30) | 0.148 |
| **Phe** | ≤8 years | 1.06 (1.00, 1.14) | 0.069 | 1.23 (1.01, 1.50) | 0.035 |
|  | >8 years | 1.04 (0.98, 1.11) | 0.166 | 1.18 (0.99, 1.39) | 0.058 |
| **Tyr** | ≤8 years | 1.00 (0.94, 1.06) | 0.933 | 1.06 (0.89, 1.26) | 0.507 |
|  | >8 years | 1.01 (0.96, 1.05) | 0.770 | 1.10 (0.96, 1.25) | 0.160 |
| **Trp** | ≤8 years | 1.04 (0.98, 1.11) | 0.213 | 1.19 (0.98, 1.43) | 0.076 |
|  | >8 years | 1.04 (0.98, 1.10) | 0.249 | 1.17 (0.99, 1.38) | 0.070 |

**Note.**  Values are HRs and 95% CIs for incident hypertension. Per 1-SD increment indicates the HR associated with a 1-standard-deviation increase in energy-adjusted aromatic amino acid intake. For quartile analyses, Q1 was used as the reference group. Models were adjusted for age, sex, urban residence, income level, education level, history of diabetes, myocardial infarction, stroke, smoking status, alcohol drinking, baseline systolic blood pressure, BMI category, abdominal obesity, physical activity level, total energy intake, sodium intake, and potassium intake, with household identifier included as a random intercept. Missing covariates were handled using multiple imputation by chained equations, and estimates were pooled across 20 imputed datasets using Rubin’s rules. AAA, aromatic amino acids; Phe, phenylalanine; Tyr, tyrosine; Trp, tryptophan; HR, hazard ratio; CI, confidence interval; BMI, body mass index.

**Supplementary Table S9. Province-adjusted sensitivity analysis of dietary aromatic amino acid intake and incident hypertension**

| **Exposure** | **Q1** | **Q2 HR (95% CI)** | **Q3 HR (95% CI)** | **Q4 HR (95% CI)** | **P for trend** | **Per 1-SD increment HR (95% CI)** | **P value** |
| --- | --- | --- | --- | --- | --- | --- | --- |
| **AAA** | 1.00  (reference) | 1.00 (0.91–1.11) | 1.08 (0.97–1.20) | 1.10 (0.97–1.24) | 0.081 | 1.02 (0.98–1.07) | 0.298 |
| **Phe** | **1.00 (reference)** | **1.00 (0.91–1.10)** | **1.11 (1.00–1.24)** | **1.19 (1.05–1.36)** | **0.003** | **1.06 (1.01–1.11)** | **0.020** |
| **Tyr** | 1.00 (reference) | 1.00 (0.91–1.11) | 0.97 (0.87–1.09) | 1.00 (0.90–1.12) | 0.972 | 0.98 (0.94–1.02) | 0.286 |
| **Trp** | **1.00 (reference)** | **1.17 (1.06–1.30)** | **1.17 (1.04–1.31)** | **1.37 (1.20–1.56)** | **<0.001** | **1.10 (1.05–1.16)** | **<0.001** |

**Note.** Values are HRs (95% CIs) from mixed-effects Cox models of 10,087 participants and 3,531 incident hypertension cases; Q1 was the reference. Exposures were energy-adjusted (g/1,000 kcal), and per 1-SD estimates represent a 1-SD increase in intake. P for trend was calculated using quartile median intakes; the final P value corresponds to the per 1-SD estimate. Model 3 adjusted for age, sex, urban/rural residence, income, education, diabetes, myocardial infarction, stroke, smoking, alcohol consumption, baseline systolic blood pressure, BMI category, abdominal obesity, physical activity, energy, sodium, and potassium intake; province was additionally included as a categorical covariate, with a household random intercept. Estimates were pooled across 20 multiply imputed datasets. AAA, aromatic amino acids; Phe, phenylalanine; Tyr, tyrosine; Trp, tryptophan; HR, hazard ratio; CI, confidence interval; SD, standard deviation; BMI, body mass index.

**Supplementary Table S10. Missingness of baseline covariates and associations with dietary aromatic amino acid intake and incident hypertension**

| **Covariate** | **Missing n (%)** | **P for AAA** | **P for Phe** | **P for Tyr** | **P for Trp** | **P for outcome** |
| --- | --- | --- | --- | --- | --- | --- |
| Age | 0 (0.0) | — | — | — | — | — |
| Sex | 0 (0.0) | — | — | — | — | — |
| Income level | 1,824 (18.1) | <0.001 | <0.001 | 0.247 | <0.001 | <0.001 |
| Education level | 81 (0.8) | 0.154 | 0.002 | 0.105 | <0.001 | 0.433 |
| Urban/rural residence | 0 (0.0) | — | — | — | — | — |
| Diabetes | 0 (0.0) | — | — | — | — | — |
| Myocardial infarction | 0 (0.0) | — | — | — | — | — |
| Stroke | 0 (0.0) | — | — | — | — | — |
| Smoking | 22 (0.2) | 0.577 | 0.879 | 0.295 | 0.589 | 0.754 |
| Alcohol use | 90 (0.9) | 0.005 | <0.001 | 0.468 | <0.001 | 0.740 |
| Baseline systolic blood pressure | 0 (0.0) | — | — | — | — | — |
| BMI category | 52 (0.5) | 0.500 | 0.447 | 0.480 | 0.878 | 0.601 |
| Abdominal obesity | 0 (0.0) | — | — | — | — | — |
| Physical activity | 17 (0.2) | 0.527 | 0.793 | 0.368 | 0.414 | 0.629 |
| Total energy intake | 0 (0.0) | — | — | — | — | — |
| Sodium intake | 0 (0.0) | — | — | — | — | — |
| Potassium intake | 0 (0.0) | — | — | — | — | — |
| **Any baseline covariate** | **2,015 (20.0)** | **<0.001** | **<0.001** | **0.173** | **<0.001** | **<0.001** |

**Note.** Missingness was assessed at each participant’s first eligible observation before multiple imputation (N = 10,087). P values are from separate univariable logistic regressions with covariate missingness as the dependent variable and each energy-adjusted dietary exposure (per 1-SD increment) or incident hypertension status as the independent variable. These analyses are descriptive and unadjusted; — indicates no missing observations. AAA, aromatic amino acids; Phe, phenylalanine; Tyr, tyrosine; Trp, tryptophan.

**Supplementary Table S11. Schoenfeld residual diagnostics of aromatic amino acid intake and incident hypertension**

| **Data column** | **Exposure test χ² (df)** | **P value** | **Global test χ² (df)** | **P value** |
| --- | --- | --- | --- | --- |
| **AAA** | 1.482 (1) | 0.223 | 29.750 (24) | 0.193 |
| **Phe** | 2.464 (1) | 0.116 | 29.315 (24) | 0.208 |
| **Tyr** | 0.00149 (1) | 0.969 | 29.932 (24) | 0.186 |
| **Trp** | 1.143(1) | 0.285 | 29.413 (24) | 0.205 |

**Note.** Schoenfeld residuals were used to assess the proportional hazards assumption for aromatic amino acid intake and incident hypertension. AAA, aromatic amino acids; Phe, phenylalanine; Tyr, tyrosine; Trp, tryptophan.

**Supplementary Table S12. Interaction between plant- and animal-derived aromatic amino acid intakes and incident hypertension**

| **Exposure** | **Interaction HR (95% CI)** | **P for interaction** |
| --- | --- | --- |
| **AAA** | 1.01 (0.97–1.06) | 0.536 |
| **Phe** | 1.01 (0.96–1.05) | 0.771 |
| **Tyr** | 1.02 (0.97–1.06) | 0.482 |
| **Trp** | 1.01 (0.97–1.06) | 0.644 |

**Note.** Plant- and animal-derived intakes were centered and standardized using their respective baseline SDs. Interaction HRs are exponentiated coefficients for their product terms; P values are two-sided Wald tests pooled across 20 imputed datasets using Rubin’s rules. Models included the covariates in Model 3 and a household random intercept. All analyses included 10,087 participants and 3,531 incident hypertension cases. These tests do not estimate substitution effects. AAA, aromatic amino acids; Phe, phenylalanine; Tyr, tyrosine; Trp, tryptophan; HR, hazard ratio; CI, confidence interval; SD, standard deviation.

**Supplementary Figure S1. Spearman correlation heatmap of total and source-specific aromatic amino acid intakes and total protein intake.**

The heatmap shows Spearman correlation coefficients among energy-adjusted intakes of total AAA, Phe, Tyr, and Trp; plant-derived and animal-derived AAA, Phe, Tyr, and Trp; and total protein. Total protein intake was also energy-adjusted and expressed as g/1000 kcal for this analysis. Correlations were calculated using pairwise complete observations. AAA, aromatic amino acids; Phe, phenylalanine; Tyr, tyrosine; Trp, tryptophan.

**Supplementary Methods.**

**Construction and harmonization of the dietary amino acid database**

Dietary exposure assessment was based on six China Health and Nutrition Survey dietary waves with available dietary data: 1997, 2000, 2004, 2006, 2009, and 2011. Individual food-level intake data from 3 consecutive 24-h dietary recalls were linked to the corresponding Chinese Food Composition Tables to estimate dietary nutrient and amino acid intakes. The 1991 Food Composition Table was applied to the 1997 and 2000 dietary waves, and the 2002/2004 Food Composition Tables were applied to the 2004, 2006, 2009, and 2011 waves.

Food items were standardized according to food name, food code, edible portion, and major food category. Food codes and food-group assignments were harmonized across Food Composition Table versions. Ambiguous code mappings and food-group classifications were manually reviewed against the original food descriptions and the corresponding Food Composition Table categories. Missing amino acid composition values were then handled according to the rule described below.

**Imputation of missing amino acid composition values**

Available Phe, Tyr, and Trp composition values were retained. For each missing amino acid value, the arithmetic mean of nonmissing values among foods within the same food-code category and harmonized major food group in the corresponding Chinese Food Composition Table was assigned programmatically. For the 1991 Food Composition Table, which was applied to the 1997 and 2000 dietary waves, food categories were identified using the category code. For the 2002/2004 Food Composition Tables, which were applied to the 2004, 2006, 2009, and 2011 waves, categories were identified using the major code derived from the standardized food code.

When no measured amino acid value was available for a food classified in a predefined low-amino-acid category, including alcoholic beverages, sugars, oils/fats, and seasonings, the value was assigned as zero. Mixed foods were classified as mixed/other according to the harmonized food-code structure and were processed using the same category- and food-group-based procedure; they were not disaggregated or assigned amino acid values according to a presumed dominant ingredient. Manual review was limited to food-code harmonization and food-group classification, whereas replacement values were calculated programmatically at the food-composition level. Individual-level dietary exposures were not imputed.

Total AAA content was calculated as the sum of Phe, Tyr, and Trp. Participant-level daily intakes of Phe, Tyr, Trp, and total AAA were calculated by multiplying the amount of each food consumed by its corresponding amino acid content and summing across all foods consumed during the 3-day dietary assessment period. Average daily intakes were used in the analyses.

**Energy adjustment and exposure construction**

To account for differences in total energy intake, total AAA and individual amino acid intakes were energy-adjusted using the nutrient density method and expressed as g/1000 kcal. The primary dietary exposures were total AAA, Phe, Tyr, and Trp, analyzed both as quartiles and as continuous variables per 1-SD increment. For the main analysis, baseline dietary intake was defined using the first eligible dietary assessment for each participant. For sensitivity analysis using cumulative average intake, repeated dietary measurements from available survey waves before each follow-up interval were used to calculate updated cumulative average intakes, thereby better reflecting long-term habitual intake and reducing within-person variation.

**Food-source classification**

All foods contributing to amino acid intake were classified into major food groups according to the Chinese Food Composition Tables and harmonized CHNS food-code structure. The major food groups included cereals/grains; soybeans, legumes, and related products; nuts and seeds; vegetables; fruits; tubers and starches; red and processed meat; poultry; fish and seafood; animal offal; eggs; milk and dairy products; and other foods. These food groups were further categorized into three broad source types: plant-derived, animal-derived, and mixed/other-derived sources.

Plant-derived sources included cereals/grains, soybeans/legumes and related products, nuts/seeds, vegetables, fruits, and tubers/starches. Animal-derived sources included red and processed meat, poultry, fish/seafood, animal offal, eggs, and milk/dairy products. Mixed/other-derived sources included mixed dishes, foods with unclear or heterogeneous ingredients, and food items that could not be assigned exclusively to plant- or animal-derived categories. This category was retained in descriptive source-contribution analyses but was not included in mutual source-specific models because of its small contribution and heterogeneous composition.

**Calculation of source-specific aromatic amino acid intakes**

For each participant, source-specific intakes of total AAA, Phe, Tyr, and Trp were calculated by summing amino acid contributions from all foods within each source category. For example, plant-derived Phe intake was calculated as the sum of Phe contributed by all plant-derived foods consumed during the dietary assessment period. Total source contributions were calculated as the percentage of each amino acid intake derived from each major food group or source category. For food-source contribution analyses, percentages were averaged across participants to describe the mean proportional contribution of each food group or source category.

For source-specific association analyses, plant-derived, animal-derived, and mixed/other-derived amino acid intakes were first examined in single-source models. In mutual models, plant-derived and animal-derived amino acid intakes were included simultaneously to evaluate whether associations differed by dietary source after accounting for the corresponding intake from the other major source category. Mixed/other-derived intakes were not included in mutual models because they contributed only a small proportion of total intake and represented a heterogeneous group of foods. All source-specific amino acid exposures were energy-adjusted and modeled per 1-SD increment.

**Quality control**

Several quality-control procedures were applied to ensure consistency of the dietary amino acid variables. First, food-code matching and amino acid assignments were checked across survey waves to minimize inconsistencies caused by changes in Food Composition Table versions. Second, the calculated participant-level intakes of total AAA, Phe, Tyr, and Trp were examined for implausible values and compared with total protein intake and total energy intake. Third, the expected relationship among amino acid components was checked, with total AAA equal to the sum of Phe, Tyr, and Trp. Fourth, food-source-specific intakes were checked to ensure that the sum of plant-derived, animal-derived, and mixed/other-derived intakes matched total intake for each corresponding amino acid exposure. Finally, descriptive distributions and correlation patterns of total and source-specific amino acid intakes were examined before association analyses.
